# Effects of *Escherichia coli* Nissle 1917 on the Porcine Gut Microbiota, Intestinal Epithelium and Immune System in Early Life

**DOI:** 10.3389/fmicb.2022.842437

**Published:** 2022-02-25

**Authors:** Mirelle Geervliet, Hugo de Vries, Christine A. Jansen, Victor P. M. G. Rutten, Hubèrt van Hees, Caifang Wen, Kerstin Skovgaard, Giacomo Antonello, Huub F. J. Savelkoul, Hauke Smidt, Edwin Tijhaar, Jerry M. Wells

**Affiliations:** ^1^Cell Biology and Immunology Group, Wageningen University & Research, Wageningen, Netherlands; ^2^Host-Microbe Interactomics Group, Wageningen University & Research, Wageningen, Netherlands; ^3^Laboratory of Microbiology, Wageningen University & Research, Wageningen, Netherlands; ^4^Division of Infectious Diseases and Immunology, Department of Biomolecular Health Sciences, Faculty of Veterinary Medicine, Utrecht University, Utrecht, Netherlands; ^5^Department of Veterinary Tropical Diseases, Faculty of Veterinary Science, University of Pretoria, Pretoria, South Africa; ^6^Research and Development, Trouw Nutrition, Amersfoort, Netherlands; ^7^Department of Biotechnology and Biomedicine, Technical University of Denmark, Lyngby, Denmark; ^8^Department of Cellular, Computational and Integrative Biology, University of Trento, Trento, Italy

**Keywords:** *E. coli* nissle 1917, porcine, early life, probiotic, immune system, gut microbiota, immunomodulation, gastrointestinal tract

## Abstract

Early in life and particularly around weaning, piglets are susceptible to infections because of abrupt social, environmental, and dietary changes. Dietary interventions with probiotic bacteria have gained popularity because of the increased awareness of the direct link between diet and health. In this study, piglets received the probiotic strain *Escherichia coli* Nissle 1917 (EcN) or a control treatment perorally from day 2 after birth until 2 weeks post-weaning. To investigate spatio-temporal effects of EcN on the gut microbiota composition, intestinal epithelial gene expression and immune system, feces, digesta, blood, scraping material and mesenteric lymph node tissue were collected at different time points. In addition, oral vaccinations against *Salmonella enterica* serovar Typhimurium were administered on days 21 and 45 of the study to assess the immunocompetence. EcN-treated pigs showed a reduced diversity of taxa within the phylum Proteobacteria and a lower relative abundance of taxa within the genus *Treponema* during the pre-weaning period. Moreover, EcN induced T cell proliferation and Natural Killer cell activation in blood and enhanced IL-10 production in *ex vivo* stimulated mesenteric lymph node cells, the latter pointing toward a more regulatory or anti-inflammatory state of the local gut-associated immune system. These outcomes were primarily observed pre-weaning. No significant differences were observed between the treatment groups with regards to body weight, epithelial gene expression, and immune response upon vaccination. Differences observed during the post-weaning period between the treatment groups were modest. Overall, this study demonstrates that the pre-weaning period offers a ‘window of opportunity’ to modulate the porcine gut microbiota and immune system through dietary interventions such as EcN supplementation.

## Introduction

Abrupt social, environmental, and dietary shifts associated with weaning lead to transient lower feed intake, activation of the hypothalamic-pituitary-adrenal (HPA) axis, increased intestinal permeability, diarrhea, and increased abundance of *Enterobacteriaceae* in the gut (Trevisi et al., 2021). These physiological responses increase susceptibility to viral and bacterial pathogens (e.g., enterotoxigenic *Escherichia coli* or ETEC) and inflict potential welfare issues over the following weeks. From the 1950’s onwards, the addition of antibiotics to pig feed has been used to combat these problems and to improve the growth of young pigs and the reproductive performance of sows (Li, 2017). However, the use of antibiotics increases the selective pressure on intestinal bacteria, resulting in antimicrobial resistance (AMR) and hence increased risk of transmission of antibiotic-resistant zoonotic pathogens to humans. In 2006, the EU prohibited the use of antibiotics as growth promoters in livestock to reduce the spread of antimicrobial resistance. Since then, other countries have prohibited the use of medically important antibiotic growth promotors (AGPs), but this is not the case in all major livestock producing countries (Kirchhelle, 2018). Effective alternative strategies in a One Health setting that support a healthy development and growth of piglets would help drive the changes needed to reduce AMR worldwide.

Probiotics are becoming increasingly popular as alternatives to AGPs (Bajagai et al., 2016). However, research outcomes in this field have been largely inconsistent, and there is still a need to optimize such probiotic strategies (Barba-Vidal et al., 2019). One of the oldest well-studied probiotics is the Gram-negative bacterial *E. coli* strain Nissle 1917 (EcN), initially isolated by Alfred Nissle during the First World War from a healthy soldier during an outbreak of diarrhea (Blum et al., 1995). Since its discovery, EcN has been used to treat intestinal diseases such as acute diarrhea, ulcerative colitis, Crohn’s disease and constipation in humans (Sonnenborn and Schulze, 2009). Clinical studies showed that oral administration of EcN attenuates acute diarrhea in infants and toddlers, and prevents diarrhea in neonatal calves (Von Buenau et al., 2005; Henker et al., 2008).

The beneficial effects of EcN are considered to be mediated through production of anti-microbial colicins and microcins, competition with pathobionts and pathogens for nutrients and adhesion sites, and immunomodulatory activities (Patzer et al., 2003; Barth et al., 2009; Jacobi and Malfertheiner, 2011; Deriu et al., 2013; Sassone-Corsi et al., 2016; Wassenaar, 2016). In humans, intestinal inflammation leads to a bloom of *Enterobacteriaceae* and increased abundance of B2 phylotype *E. coli* that adhere to the intestine and elicit damage through hemolysin production, which further promotes mucosal inflammation and intestinal barrier disruption. EcN is of the same phylotype as these adherent and invasive *E. coli* (AIEC) and can compete for adherence without affecting gut integrity (Mirsepasi-Lauridsen et al., 2016).

Studies with EcN and intestinal cell lines report increased expression of tight junction protein zonula occludens 2 (ZO-2) (Zyrek et al., 2007), induction of β-defensin-2 expression through NF-kB- and AP-1-mediated signaling (Wehkamp et al., 2004), and production of interleukin (IL)-8 (Lammers et al., 2002). Moreover, EcN has shown to modulate the activity of cells of the porcine innate and adaptive immune systems (Vlasova et al., 2016; Michael et al., 2021a). However, the exact molecular mechanisms are still under investigation. *In vitro*, EcN enhances dendritic cell (DC) maturation and cytokine production, and induces activation, cell cycling and expansion of human γδ T cells (Guzy et al., 2008; Geervliet et al., 2020). Moreover, several studies in different animal species demonstrated that EcN alters host cytokine responses upon stimulation, particularly by inducing high levels of the anti-inflammatory cytokine IL-10 (Cross et al., 2004; Güttsches et al., 2012; Kandasamy et al., 2016). These immunomodulatory effects are considered to be primarily induced through a Toll-like receptor 4 (TLR-4) dependent signaling pathway (Grabig et al., 2006; Adam et al., 2010). *In vivo* studies using gnotobiotic pigs showed that colonization with EcN mediated greater protection against human rotavirus challenge than *Lactobacillus rhamnosus* GG (LGG), possibly due to stimulation of the innate immune system and activation of the DC-IL-12-NK immune axis (Kandasamy et al., 2016; Vlasova et al., 2016).

Although EcN has been studied for over a century (Wassenaar, 2016), little is published about the *in vivo* effects of EcN on the gut microbiota, gut epithelial barrier and immune system of pigs. Moreover, there is limited data available on the presence, abundance and persistence of EcN in the gastrointestinal tract (GIT) of conventional pigs. The aim of this research was therefore to determine whether peroral administration of EcN to postnatal piglets would impact on the development of the porcine gut microbiota, intestinal barrier function and the innate and adaptive arms of the immune system. A more mature or established gastrointestinal system could ultimately facilitate the establishment of a vigilant immune system that counteracts pathogens during the weaning and post-weaning period.

## Materials and Methods

### Ethics Statement and Study Design

This study complied with the European directive 2010/63/EU on the protection of animals used for scientific purposes, and was conducted in accordance with the Dutch law on animal experimentation and ethical requirements. The project was approved and licensed (Permit Number: AVD1040020173948) by the Dutch Central Authority for Scientific Procedures on Animals (CCD), and all associated protocols were approved by the Animal Experimentation Committee of Wageningen University & Research (Wageningen, Netherlands). Pigs were euthanized by intravenous injection with 20% sodium pentobarbital (Euthasol^®^), followed by immediate exsanguination. Pigs were euthanized according to Good Veterinary Practice (GVP), and all efforts were made to minimize suffering or discomfort.

**FIGURE 1:**
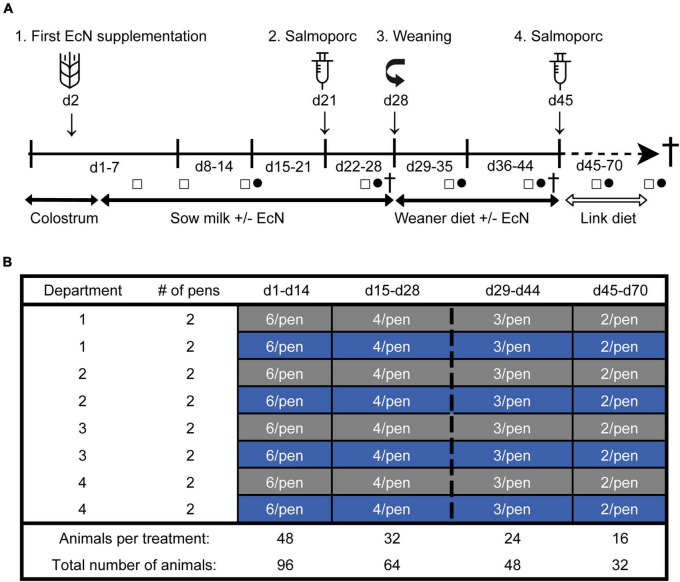
Timeline of the study **(A)**. From day 2 until day 44, piglets received an oral dose of EcN or were sham-dosed with tap water (Control) every other day (1). A subset of piglets was weaned on day 28 (3). All pigs received an oral vaccination (Salmoporc^®^) against *Salmonella enterica* serovar Typhimurium on days 21 [primary vaccination; (2)] and 45 [booster vaccination; (4)]. Fecal sample collection was performed on days 4, 8, 14, 26, 35, 43, 59, and 69 (squares; □) and blood samples were taken on days 14, 26, 35, 43, 59, and 69 (circles; •). Subsets of animals were sacrificed on days 27, 44, and 70 (cross; †). Schematic representation of the experimental design showing the remaining number of animals per treatment over time as determined by deselection or dissection of animals **(B)**. The control and the EcN groups are presented as gray and blue, respectively.

The study was performed at the Swine Research Center (Trouw Nutrition, Sint Anthonis, Netherlands) and included 33 sows (Hypor Libra) and their litters (Maxter × Hypor Libra sow). To reduce genetic variation in the litters, sperm from a single boar was used to inseminate all sows. Shortly after birth, all piglets received an intramuscular injection of iron, an ear tag for identification, and their tails were docked. In addition, their sex and birth weight were recorded. Approximately 24 h after birth, 192 female piglets were selected and cross-fostered to minimize possible confounding effects, including the day of birth, weight at birth, litter size, sow parity and genetic background. At cross-fostering, from the original 192 piglets, 96 piglets were randomly allocated to either the control- or treatment (EcN) group, resulting in 16 pens (pen is the experimental unit) with one sow and six piglets each (eight pens per treatment group). Pens were distributed over four farrowing rooms and balanced for treatment. Male and non-experimental female piglets were housed together with the focal piglets by equally dividing them over the pens. A schematic representation of the experimental design and the study timeline is presented in Figure 1. All piglets were housed together with their fostering mother until weaning (approximately day 28), in a room with a computer-controlled climate system. To keep the floors sufficiently dry in the farrowing rooms, calcium carbonate-based powder (Power-Cal^®^, Power-Cal, Netherlands) was added to all pen floors in the first few days post-farrowing. In addition, various measures were taken to prevent cross-contamination of EcN between the farrowing pens, including pen separators, separate equipment (e.g., boots and overalls), and hygiene protocols. A weaner diet was fed 3 days before weaning (from days 25 to 27) to allow piglets to become acquainted with the consumption of solid feed (de Vries et al., 2020). At weaning, a subset of pigs (48 pigs, 3 per pen) was randomly selected and reallocated to a nursery facility. Pigs that received medication or suffered from leg/claw injuries or growth retardation were excluded from this selection. All pigs received a weaner diet from day 28 until day 44, followed by a nursery diet until the end of the study (de Vries et al., 2020). During the entire post-weaning period all piglets had access to solid feed and water *ad libitum*. On days 21 and 45 all piglets perorally received a *Salmonella enterica* serovar Typhimurium (Salmoporc^®^) vaccination and on days 27, 44, and 70, sixteen piglets were sacrificed (eight per treatment, at each time point).

### Experimental Procedures

#### EcN Administration and Oral Vaccination With Salmoporc^®^

*Escherichia coli* Nissle 1917 (Ponsocol^®^) was kindly provided by Ardeypharm GmbH (Herdecke, Germany) and stored until use at 4°C. From day 2 onwards, pigs perorally received either a control treatment (tap water) or EcN (10^9^ CFU/administration) every other day until day 44 of the study. EcN was administered at increasing volume over time ranging from 1 to 5 mL, using disposable syringes with a volume of 2 and 5 mL (Discardit II, BD). The sham-dosed control piglets similarly received water. To prepare EcN at the required concentration, Ponsocol^®^ vials (containing 10^8^ CFU/mL) were gently agitated and pooled, followed by centrifugation at 5,200 × g for 5 min. The bacterial pellet was resuspended to the required concentration using excess supernatant. CFU counts of Ponsocol and the final solution were confirmed by plating serial dilutions on lysogeny broth (LB), followed by counting.

To investigate the effect of EcN on the immune response to vaccination, all pigs were vaccinated against *Salmonella enterica* serovar Typhimurium (Salmoporc^®^ STM, lot number 0270617, IDT Biologika GmbH) on days 21 (primary vaccination) and 45 (booster vaccination). This oral live-attenuated vaccine is registered for pigs to inhibit bacterial colonization, excretion, and clinical symptoms of *Salmonella enterica* serovar Typhimurium. The vaccine was reconstituted in water before oral administration according to the manufacturer’s instructions.

#### Blood and Fecal Sampling

For both fecal- and blood samples, piglets were sampled in random order and weighed shortly before sample collection. Fecal samples were collected in cryotubes on days 4, 8, 14, 26, 35, 43, 59, and 69 by gentle rectal stimulation with cotton swabs (PurFlock Ultra, Puritan) pre-wetted with sterile water. The cryotubes were immediately placed on dry ice and stored at −80°C until further processing.

Blood samples were collected from the jugular vein of sixteen animals on days 14, 26, 43, and 69 using Serum Gel tubes (S-Monovette^®^, Sarstedt) that were centrifuged at 2,500 × g for 10 min to separate serum. Serum was stored at −20°C until further use. Blood samples for flow cytometry were collected in Sodium heparin tubes (S-Monovette^®^, Sarstedt) and stored at room temperature (RT) until further processing.

#### Dissection

Immediately after euthanasia and exsanguination of a pig, the ileocecal mesenteric lymph node (MLN) was removed and stored on ice in cold (4°C) RPMI 1640 Medium with GlutaMAX™ supplement (Gibco^®^), 2 mM L-Glutamine (Gibco^®^), and 10% fetal calf serum (FCS, Gibco^®^). Next, the GIT was removed from the abdominal cavity, then the jejunum, ileum, cecum and colon were identified and segmented accordingly (Supplementary Figure 1). Digesta samples from each gastrointestinal segment were taken by gently squeezing the content of a 40 cm segment into a plastic container (see Supplementary Figure 1 for a visual overview). Next, the digesta were homogenized using a sterile spatula, and approximately 1 g was aliquoted and stored in sterile cryogenic vials. The vials were immediately snap-frozen on dry ice and stored at −80°C until further processing. Leftover digesta from each gastrointestinal segment were mixed with water for pH measurement using a pH meter. Mucosal scrapings of the ileal and colonic gut epithelium were collected by taking 5 cm long gut segments adjacent and proximal to the gut segments used for digesta sampling (Supplementary Figure 1). First, the 5 cm segment was cut open longitudinally and cleaned using sterile Phosphate Buffered Saline (PBS). Then, mucosae were collected by scraping using a scalpel. Care was taken not to include muscular tissue. Mucosal scrapings were directly stored in Snaplock tubes containing RNA-later (Sigma-Aldrich, St. Louis, MO, United States), placed on dry ice, and stored at −80°C.

### Measurements

#### Quantification of EcN by qPCR

A qPCR with primers specific to EcN (Table 1) was used to determine the relative abundance of EcN in fecal samples. All qPCR analyses were performed in triplicate in a reaction volume of 10 μL, using Hard-Shell^®^ 384-Well PCR plates (Bio-Rad). The reaction mixture contained 2x iQ SybrGreen Supermix (Bio-Rad Laboratories B.V., Lunteren, Netherlands), 200 nM of each primer (Table 1), and 2 μL of the DNA template (1 ng/μL). The amplification program consisted of an initial denaturation at 94°C for 10 min followed by 39 cycles of 94°C for 20 s, 60°C for 30 s, and 72°C for 30 s using a CFX384TM thermocycler (Bio-Rad Laboratories B.V., Lunteren, Netherlands). The fluorescent products were detected at the last step of each cycle. Following amplification, melting temperature analysis of PCR products was performed to determine the specificity of the PCR. The melting curves were obtained by slow heating with 0.5°C/min increments from 60 to 95°C with continuous fluorescence reading. For both EcN-specific and total bacterial primers, standard curves for the qPCR assays were prepared with tenfold serially diluted EcN genomic DNA as a template. The EcN qPCR was performed on a subset of the fecal and digesta samples to validate the EcN-specific ASV in the NGS dataset.t

**TABLE 1:** Primers used for the quantification of EcN.

Name	Target	Sequence (5′–3′)	Annealing temp (°C)	Length of PCR product (bp)	References
BACT1369f	16S rRNA region	CGG TGA ATA CGT TCY CGG	60	123	van Lingen et al., 2017
BACT1492r	16S rRNA region	GGW TAC CTT GTT ACG ACT T	60		
EcN_for	EcN chromosome	GAA GAA ATT GAC GCA CCC C	60	110	Reister et al., 2014
EcN_rev	EcN chromosome	CGT GCA AGA CAT GGA GAG AC	60		

#### Determination of Microbiota Composition

A personalized Maxwell 16 Total RNA protocol (Promega Corp., Madison, WI, United States) was used to extract DNA from fecal and digesta samples. DNA extraction and further processing were performed exclusively on samples from animals that were dissected during the study. In addition to samples from this study, synthetic mock communities, blank water negative controls and EcN spiked samples were included in the sequencing libraries. Spiked samples (spk1 – spk5) were generated by mixing 0.05 mL 10-fold serial dilutions of Ponsocol^®^ to 0.05 g of fecal material that was tested negative for the presence of EcN (Supplementary Figure 2). This resulted in five spiked fecal samples, ranging from 10^6^ CFU/mL of EcN in the most diluted sample (spk1) to 10^10^ CFU/mL of EcN in the most concentrated sample (spk5).

For PCR amplification, barcoded primers directed to the V4 region of the bacterial and archaeal 16S rRNA gene were used, namely EMP_515F (5′-GTGYCAGCMGCCGCGGTAA), with linker ‘GT,’ and EMP_806R (5′-GGACTACNVGGGTWTCTAAT), with linker ‘CC.’ PCR reactions were done in duplicate for digesta samples and in triplicate for fecal samples. The amplification program included 30 s initial denaturation at 98°C for 10 s, annealing at 50°C for 10 s, elongation at 72°C for 10 s, and a final extension at 72°C for 7 min. For a detailed description of the DNA extraction protocol and the PCR protocol, see de Vries et al. (2020). DNA extraction from jejunum digesta samples occasionally resulted in low DNA yields (i.e., <1 ng/μL). For this reason, the Qubit dsDNA BR Assay Kit (Thermo Fisher Scientific, Waltham, MA, United States) was used to measure the DNA concentration of all jejunum samples instead of a NanoDrop ND-1000 spectrophotometer (NanoDrop Technologies Inc., Wilmington, DE, United States). The latter was used for all ileum, cecal and fecal samples. An overview of DNA concentrations and quality readings can be found in Supplementary File 1. For the purification of PCR products, the HighPrep PCR kit (MagBio Genomics Inc., Gaithersburg, MD, United States) was used. DNA concentrations were measured with the Qubit dsDNA BR Assay Kit. The amplicon pool was generated by combining 200 ng of PCR amplified DNA from each sample and then concentrated to a final volume of approximately 20 μL using the HighPrep PCR kit. The Qubit dsDNA BR Assay Kit was used to measure the DNA concentration of the amplicon pool, and libraries were sent for Illumina HiSeq sequencing (Sequence mode used: NovaSeq 6000 S2 PE150 XP, Eurofins Genomics, Ebersberg, Germany). Amplicon sequence data were processed and analyzed using NG-Tax 2.0 (Poncheewin et al., 2019), and annotated using the SILVA (release 132) database (Quast et al., 2012). The resulting dataset, containing microbiota composition data, will be referred to as the NGS dataset and contains taxonomic assignments up to the level of amplicon sequence variants (ASVs) that each has a unique ASV number. Each ASV is aimed to represent one bacterial species, and, where possible, were annotated at the genus level.

#### Gastrointestinal Mucosal Gene Expression Analysis

Mucosal scrapings of the epithelium were thawed on ice and excess RNA-later was removed. Then, a small portion (approximately 3 by 3 mm) was taken using a scalpel and tweezers, and added to 500 μL RLT (RNeasy lysis) buffer (Qiagen, Hilden, Germany). The sample was homogenized for 90 s using an Ultra-Turrax IKA T-10 Basic (IKA-Werke GmbH, Staufen, Germany). After homogenization of each sample, the shaft of the Ultra-Turrax IKA T-10 Basic was cleaned twice by running the device in a tube containing an excessive amount of fresh PBS. This was done to prevent cross-contamination of samples. Next, 100 μL of homogenate was thoroughly mixed with 600 μL ice-cold RLT buffer. Then, 700 μL of 70% ethanol was added, followed by thorough mixing. RNA was isolated using the RNeasy Mini Kit (Qiagen, Hilden, Germany), according to manufacturer’s instructions including DNAse treatment to remove host DNA. The concentration of purified RNA was measured using the Qubit BR RNA Assay Kit (Thermo Fisher Scientific), and the integrity value of each RNA sample was assessed using the Qsep 100 protocol (GC Biotech, Waddinxveen, Netherlands). The RNA Quality Number was higher than 8.0 for all samples. After isolation, RNA was directly stored at −80°C. The QuantiTect Reverse Transcription Kit (Qiagen, Hilden, Germany) was used for cDNA synthesis (in duplicate) with 500 ng of RNA, and included the additional removal of genomic DNA (gDNA Wipeout Buffer). Genes of interest were chosen based on functionality within the mucosal barrier and functionality related to local and systemic immune responses. Distribution and lengths of introns and exons within genes of interest were found using Ensembl^1^. Primers were designed to span intron/exon boundaries using Primer3 version 0.4.0^2^ if possible, and checked for interspecies-variation using BLAST^3^. Primers were synthesized by Sigma Aldrich (Sigma-Aldrich, St. Louis, MO, United States). A total of 96 primer pairs were used for gene expression analysis. Primer sequences, corresponding genes, primer efficiency, and amplicon lengths can be found in Supplementary File 2.

For pre-amplification, 3 μL TaqMan PreAmp Master Mix (Applied Biosystems, Waltham, MA, United States), 2.5 μL 200 nM mix of each of the 96 primer pairs, 2 μL low-EDTA TE-buffer (Panreac Applichem, Darmstadt, Germany) and 2.5 μL diluted cDNA (1:10 in low-EDTA TE-buffer) was mixed and incubated at 95°C for 10 min followed by 19 cycles of 95°C for 15 s and 60°C for 4 min. The pre-amplified cDNA was treated with 16 U Exonuclease I (New England Biolabs, Ipswich, MA, United States) for 30 min at 37°C, followed by 80°C in 15 min. Prior to qPCR analysis, the pre-amplified cDNA was diluted 1:10 in low-EDTA TE-buffer. Microfluidic qPCR were performed on a Biomark HD Reader (Fluidigm, CA, United States) using the following cycle parameters: 50°C for 2 min, 95°C for 10 min followed by 35 cycles at 95°C for 15 s and 60°C for 1 min. Melting curves were generated as described in “Quantification of EcN by qPCR” after the 35 cycles of amplification. For each time point and gut segment, data were pre-processed and normalized separately using GeneEx5 Real-Time PCR Analysis software (MultiD, Göteborg, Sweden), as described earlier by Brogaard et al. (2015). For each gut segment (ileum or colon), reference genes were selected using the algorithms geNorm and NormFinder (Vandesompele et al., 2002; Andersen et al., 2004). Primer pairs that resulted in inconsistent replicates were excluded from the dataset. Paired t-tests were performed using Excel 2012 (Microsoft, Redmond, WA, United States), and a dataframe with resulting *p*-values was imported into R statistical software (version 3.6.1). *P*-values were adjusted for multiple testing using the Benjamini–Hochberg false discovery rate (fdr) correction, and resulting adjusted *p*-values (p.adjust) were used in combination with mean fold-change values (between treatments) to create volcano plots. Exclusively genes with a fold change > 2 were provided with a label and genes were considered to be statistically differentially expressed if *p*-adj < 0.05.

#### Serology

The antibody response against *Salmonella enterica* serovar Typhimurium vaccination was measured using an ELISA. The live attenuated strain of *Salmonella enterica* serovar Typhimurium (Salmoporc^®^) was grown overnight at 37°C on MacConkey agar (Sigma-Aldrich). Next, a single colony was inoculated into 2 mL of lysogeny broth medium and incubated overnight at 37°C with shaking (200 rpm). The following day, 1 mL of this overnight culture was transferred to a 50 mL tube that contained 15 mL of fresh LB medium. The 50 mL tube was then placed on a shaker (200 rpm) at 37°C under aeration until exponential growth phase (OD_600*nm*_ = 0.6 to 0.8) was reached. Bacteria were then pelleted by centrifugation at 10,000 rpm for 5 min, followed by two washes with cold PBS. Next, this bacterial suspension (2 × 10^8^ bacteria/mL, 100 μL/well) was used to coat 96 well plates (medium-binding flat bottom clear wells, Greiner Bio-One) that were incubated overnight at 4°C. The following day, the bacterial suspension was removed and bacteria still attached to the plate were fixed with 4% paraformaldehyde in PBS for 2 h at RT. Plates were then blocked overnight at RT with a blocking solution consisting of 5% milk powder (ELK, FrieslandCampina) in demi water. After overnight blocking, plates were stored at 4°C until use. Just before use, plates were washed with PBS/Tween20 (0.05%) and 100 μL of sera diluted in blocking solution (250× to determine IgG levels and 50× for IgA and IgM) were added. After 1 h of incubation at RT, plates were washed two times. Next, 100 μL horseradish peroxidase (HRP) conjugated goat anti-Porcine IgM, IgA, or IgG (Novus Biologicals) diluted 1:50,000 in blocking solution was added to the plates. After approximately 30 min, plates were washed five times and incubated with 100 μL of 3,3′,5,5′- tetramethylbenzidine (TMB) substrate solution (Enhanced K-Blue^®^, Neogen). After 15 min the reaction was stopped by adding 100 μL of stop solution (2% HCl). The optical density (OD) of the plate content was measured at 450nm (Multi-Mode Microplate Reader FilterMax F5).

#### Isolation of Immune Cells

Peripheral blood mononuclear cells (PBMCs) were isolated within 4 h using 50 mL Leucosep™ tubes (Greiner Bio-One, Alphen a/d Rijn, Netherlands) filled with 60% FICOLL-PAQUE™ Plus density-gradient according to manufacturer’s protocol. All blood samples were diluted 1:1 with PBS (containing 0.5 mM EDTA) prior to cell isolation. Any remaining red blood cells were lysed with ACK lysis buffer (Gibco^®^). To collect MLN cells, lymph nodules were cut into small pieces, gently squeezed with a syringe plunger, and passed through a sterile Falcon^®^ cell strainer (100 μm, Corning^®^) using sterile Mg^2+^ and Ca^2+^ free PBS (Lonza, Basel, Switzerland). Isolated PBMCs and MLN cells were stored overnight in PBS at 4°C until further processing.

#### Cell Stimulation Assay

Dilutions of LPS (serotype O55:B5/L2880, Sigma-Aldrich), Concanavalin A (ConA, C2010, Sigma-Aldrich), or cell culture medium only (no stimulus) were prepared the previous day and stored at 4°C overnight. The following day, plates were placed at 37°C for 30 min for temperature adjustment before adding cells. Next, PBMCs and MLN cells were seeded into 96 well clear round-bottom plates (Greiner Bio-One) at a final concentration of 1 × 10^6^ cells/200 μL. Cells were incubated with 5, 2.5, or 1.25 μg/mL of ConA, with 10, 1, or 0.1 μg/mL of LPS, or without stimuli for 24 h at 37°C (5% CO_2_). After 24 h of incubation, plates were centrifuged at 300 × *g* for 3 min, and cell culture supernatant was collected and stored in 96 well polypropylene plates (Nunc^®^, MicroWell™, Sigma-Aldrich) at −80°C until further processing. On the day of analysis, cell culture supernatant was thawed for measurement of Tumor Necrosis Factor alpha (TNFα) and Interleukin 10 (IL-10) using DuoSet ELISA kits (R&D systems, Minneapolis, MN, United States) according to the manufacturer’s instructions.

#### Flow Cytometry

The PBMCs and MLN cells were detected with DC or T lymphocyte/NK cell antibody panels (Table 2). For cell analysis, 5 × 10^6^ (DC panel) or 1 × 10^6^ (T lymphocyte/NK) PBMCs or MLN cells were transferred to 96 well polypropylene plates (Nunc^®^, MicroWell™, Sigma-Aldrich). Next, 200 μL/well FACS buffer (Mg^2+^ and Ca^2+^ free PBS; Lonza), 2 mM EDTA (Merck), 0.5% BSA fraction V (Roche) was added, and cell were washed by centrifugation at 400 × g for 3 min at 4°C. Next, the expression of markers specific for the various subsets of immune cells was determined by extracellular staining of cells with an antibody mix for 30 min on ice (in the dark), followed by one washing step with FACS buffer and two washing steps with cold PBS. Then, cells were stained with (in PBS diluted) Streptavidin-BV421 (DC panel) to detect CADM1 and Fixable viability dye eFluor™ 506 (eBioscience™) to discriminate live and dead cells. After incubation of 30 min, cells were washed with FACS buffer. To stain intracellular proteins, 100 μL Fix/Perm buffer (eBioscience™) was administered to each well, followed by a 45 min incubation at RT. Next, cells were washed three times in Perm buffer (eBioscience™), followed by incubation with an intracellular marker-specific antibody mix (Table 2) in 35 μL Perm buffer for 30 min at 4°C. Next, cells were washed two times with Perm buffer, and resuspended in 200 μL FACS buffer. Cells were measured for 300 s (DCs) or 150 s (NK cells/T cells) on the FACS CANTO II (medium flow rate). Beads (UltraComp eBeads™, Thermo Fisher Scientific) were used for single-color compensation controls. All panels were validated using Fluorescence Minus One (FMO) controls. Flow cytometry data analysis was performed using FlowJo™ software (Version 10). Identification of DCs, NK cells, and T cells was done according to previous studies (Gerner et al., 2009; Auray et al., 2016; Vreman et al., 2018), and details of the gating strategies are shown in Supplementary Figure 3 (DCs) and Supplementary Figure 4 (NK cells/T cells).

**TABLE 2:** List of antibodies used for identifying DCs, NK cells, and T cells in PBMCs and MLN cells.

Antibody	Host/isotype	Clone	Fluorochrome	Company	Dilution
**Antibody panel for the identification of DC subsets and DC**					
CD14	Mouse, IgG2b	MIL-2	FITC	Bio-Rad	1:50
CD172a	Mouse, IgG2b	74-22-15A	PE	BD Biosciences	1:40
CD4a	Mouse, IgG2b	74-12-4	PerCP-Cy5.5	BD Pharmingen™	1:320
CADM1	Chicken, IgY	3E1	Biotin	MBL	1:200
Streptav.	n/a	n/a	BV421	BD Horizon™	1:50
CD152*^a^*	Mouse, IgG2a	n/a	APC	Ancell	1:320
**Antibody panel for the identification of NK cells and T cell subsets**					
CD3ε	Mouse, IgG2a	BB23-8E6-8C8	PE-Cy™7	BD Pharmingen	1:160
CD4a	Mouse, IgG2b	74-12-4	PerCP-Cy5.5	BD Pharmingen™	1:320
CD8a	Mouse, IgG2a	76-2-11	FITC	BD Pharmingen™	1:10
FoxP3*^b^*	Rat, IgG2a	FJK-16s	Alexa Fluor^®^ 700	eBioscience™	1:20
Ki67*^b^*	Mouse, IgG1	B56	BV421	BD Horizon™	1:80
CD25*^c^*	Mouse, IgG1	K231.3B2	Purified	Bio-Rad	1:200
γδ T cells	Rat, IgG2a	MAC320	PE	BD Pharmingen™	1:20

*^a^Human CD152 (CTLA-4) murine Ig fusion protein for the identification of CD80 and CD86 on DCs.*

*^b^Antibodies against intracellular antigens.*

*^c^The ReadiLink™ Rapid iFluor™ 647 Antibody Labeling Kit (Aat Bioquest) was used to identify CD25.*

#### Colony PCR

During dissection, the ileocecal MLN was removed, rinsed with Milli-Q water, and stored in ice-cold PBS. Within 12 h after dissection, separate nodules of the MLN were passed through a cell strainer (BD Falcon™ Nylon 100 μm Cell Strainer) in a Biosafety Cabinet, and 1 mL of the MLN cell suspension was stored in an Eppendorf tube at 4°C. The next day, 100 μL of the MLN cell suspension was plated on MacConkey (MAC), incubated overnight at 37°C, and stored at 4°C for later use. A subset of red/pink colored colonies was picked, and a colony PCR (cPCR) was performed to identify EcN colonies using EcN-specific primers (see Table 1). For each reaction, 12.5 μL Onetaq buffer, 0.5 μL dNTPs (10 mM), 0.5 μL of each primer (10 μM), 0.125 μL Onetaq polymerase were combined with H_2_O to reach a final volume of 25 μL and were incubated at 95°C for 5 min followed by 35 cycles of 95°C for 45 s, 60°C for 45 s and 72°C for 45 s. Resulting amplicons were visualized using a 1% agarose gel.

### Statistical Analyses

To estimate the impact of the probiotic intervention on alpha diversity of the microbiota in pig feces, Shannon diversity and Observed Species richness were calculated for each sample at ASV resolution. In addition, Shannon diversity and Observed Species richness were similarly calculated for the phylum Proteobacteria, by first filtering out ASVs from the phylum Proteobacteria using the prune_taxa function of the phyloseq R package (McMurdie and Holmes, 2013). The Observed Species index sums the number of bacterial taxa identified in each sample, while the Shannon diversity index additionally takes into account the relative abundance of each taxon. It should be noted that rare sequences are missing from the dataset as NG-Tax 2.0 uses an abundance threshold of 0.1% for ASVs in a given sample. Shannon diversity and Observed Species richness were used in a Linear Mixed-Effects Model (i.e., Shannon ∼ Day_of_study * Treatment) to test for significant differences between treatment groups (lme function of nlme package) (Pinheiro et al., 2015). For this analysis, complete fecal sample datasets were tested, but also subsets thereof (pre- or post-weaning fecal samples exclusively). To provide additional insights into the relative abundances of ASVs within the Proteobacteria phylum, a heatmap was generated that contains all fecal samples of the pre-weaning period, with a clear separation between treatment groups. To estimate the impact of EcN on overall microbiota composition, PERMANOVA tests were performed using Bray-Curtis dissimilarities at ASV level (9,999 permutations). The homogeneity assumption was tested by calculating Bray–Curtis dispersion for each group followed by ANOVA tests. To visualize the beta diversity in fecal samples over time, a Principal Coordinates Analysis (PCoA) plot was generated using Bray-Curtis dissimilarities (Supplementary Figure 5). Beta diversity was calculated at the ASV level using Bray-Curtis dissimilarities and a PERMANOVA test was performed on all fecal samples with treatment and time point as model parameters (adonis function of the vegan R package (Shankar et al., 2017). In order to estimate the impact of EcN as a probiotic intervention on the relative abundance of specific genera in pig feces, ASV counts were first aggregated at genus level using the tax_glom function of the phyloseq R package (McMurdie and Holmes, 2013), after which read frequencies were transformed to compositional data using the transform function of the microbiome R package (Lahti and Shetty, 2017). Genera were filtered to exclusively include genera that had a relative abundance of 0.1% in at least 30% of the samples (prevalence filter). Resulting genera were tested for differential relative abundance using the Generalized Additive Models for Location, Scale and Shape (GAMLSS) with a zero-inflated beta family (function taxa.compare) with GAMLSS-BEZI as a statistical method in the metamicrobiomeR package (Ho et al., 2019). Probiotic intervention was used as the main variable for comparison and day of study and department (farrowing rooms) were used as adjusting variables. Ear tag was indicated as the identifier to enable a longitudinal approach, and “fdr” (False Discovery Rate) was chosen as the method for multiple testing adjustment (*p*.adjust < 0.05 as the threshold). For this analysis, complete fecal sample datasets as well as subsets (pre- or post-weaning fecal samples exclusively) were tested.

A Linear Mixed Model (LMM) was used to assess the immunological effects over time and the interaction between time and treatment, using R statistical software (version 3.6.2). Additionally, unpaired Student’s *t*-tests were performed to analyze the differences between the treatment groups per time point. Normality of data (Shapiro–Wilk test) and homogeneity of variances (Levene’s test) were checked prior to statistical testing. Skewness values between −2 to +2 were considered acceptable (George, 2011). Extreme outliers (indicated by R) were removed from the analysis. When normality of data or homogeneity of variances were not met, data were log-transformed but presented as untransformed means. Results with an adjusted *p*-value below 0.05 were considered statistically significant and results between 0.05 and 0.1 were considered a trend.

For a complete and detailed overview of the statistical analyses, all R-scripts, data files, and pdf files with extensive information on the performed analyses can be accessed through the following doi: 10.4121/15060177. Alternatively, these files can be found under the following Github page: https://github.com/mibwurrepo/Geervliet-et-al-2022-porcine-study-EcN.

## Results

### Spatio-Temporal Dynamics of EcN Occurrence

**FIGURE 2:**
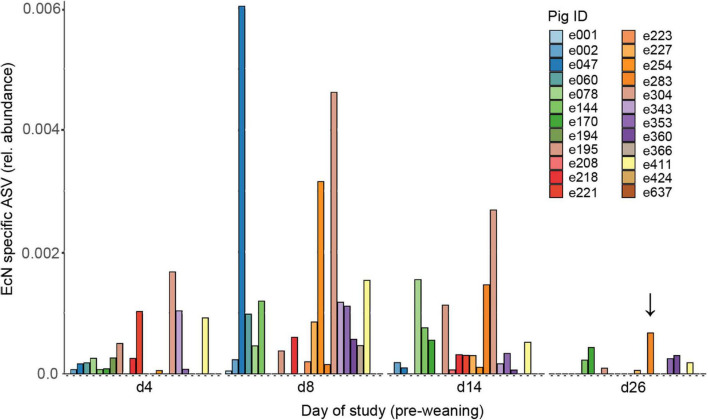
Presence of EcN in feces from EcN-treated pigs during the pre-weaning period. Data are presented as the relative abundance of the EcN-specific ASV, as calculated from the NGS dataset. Colors correspond to an individual pig (Pig ID, *n* = 24). The arrow indicates the pig (e254) from which we also found EcN in the MLN by using colony PCR. EcN was not detected in the post-weaning period and therefore only the pre-weaning data is presented.

To investigate the spatio-temporal dynamics of EcN following peroral administration, feces and digesta were collected at different time points during the study. After analyzing fecal samples that were spiked with EcN, we identified only a single EcN-specific ASV that corresponded to the amount of EcN in spiked samples (Supplementary Figure 2A). The amount of EcN identified with this EcN-specific ASV correlated well (*R*^2^ = 0.84) with the amount of EcN identified by qPCR (Supplementary Figure 2B). Therefore, the EcN-specific ASV in the NGS data was used to assess the relative abundance of EcN in all fecal samples. The relative abundance of EcN was variable among pigs in the EcN treatment group, and in only a small number of treated animals EcN was detected at all pre-weaning time points (Figure 2). EcN was not detected in digesta or feces of control animals, with the exception of one animal with a very low relative abundance of EcN (6.2 × 10^–5^) in feces on day 4 of the study. EcN could not be detected in feces of two EcN-treated pigs throughout the pre-weaning period. Peak abundances of EcN were observed on days 8 and 14 of the study, followed by a substantial decline toward day 26. Interestingly, EcN was also detected by colony PCR in the MLN of one pig on day 27, as indicated by the arrow in Supplementary Figure 6. EcN was not detected in post-weaning fecal samples, despite the fact that the treatment group still received EcN up to day 44 (data not shown). EcN was detected in jejunum, ileum, and cecum digesta in a few individuals on day 27, while on day 44 no EcN was found in any of the gut segments. Interestingly, EcN was detected in jejunal or ileal digesta of three pigs at the end of the study (day 70), which illustrates that EcN persisted in some individuals for at least 26 days after the final administration (Supplementary Figure 7). Finally, no adverse health effects were observed after peroral administration of EcN, and there were no indications that EcN had a negative effect on body weight (Supplementary Figure 8).

### Effect of EcN Treatment on the Development of Intestinal Microbiota

#### Alpha and Beta Diversity

**FIGURE 3:**
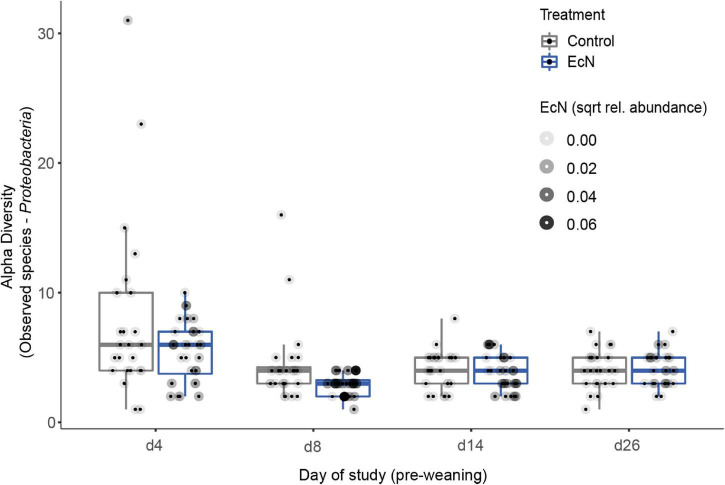
Comparison of the number of observed ASVs in the Proteobacteria phylum in fecal samples between treatments and over time (pre-weaning). Every point represents a single animal within the control group (gray box) and the treatment group (blue box). Observed Species richness values are given by sampling time point (d4–26). A significant difference in the amount of Proteobacteria ASVs was found when including only pre-weaning time points. For each sample, the relative abundance of the EcN-specific ASV is visualized by tinting the outer circle (a darker tint means a higher abundance of EcN).

To assess potential effects of EcN on microbiota composition and diversity, fecal samples were collected at multiple time points during the study (Figure 1). No significant differences in alpha diversity were observed between the EcN treatment and control groups when including all time points and all microbial taxa (Supplementary Figure 9). Similarly, no significant differences in alpha diversity were observed when only including pre-weaning time points. As ASVs within the phylum of Proteobacteria were previously reported to be affected by EcN (Šmajs et al., 2012), the alpha diversity of this phylum was investigated in more detail after removing ASVs belonging to other phyla. Interestingly, a significantly lower number of Proteobacteria ASVs were detected in EcN-treated animals during the pre-weaning period (*p* < 0.04, Figure 3). A more detailed heatmap with all Proteobacteria ASVs that includes all pre-weaning time points is presented in Supplementary Figure 10. Especially ASVs within the family of *Enterobacteriaceae* contributed to a higher number of Proteobacteria ASVs in the control group (Figure 3).

The effect of the probiotic treatment on microbiota beta diversity was significant (*p* < 0.015). In addition, a clear time effect was observed (*p* < 0.0001), which is also apparent from Supplementary Figure 5. When only including pre-weaning fecal samples, the effect of treatment was more significant (*p* < 0.005), but with a low coefficient of determination (*R*^2^ < 0.011). A statistically significant treatment effect was not observed when only post-weaning samples were included (*p* > 0.075, *R*^2^ < 0.014), which indicates that EcN had a more pronounced effect on microbial composition during the pre-weaning period.

#### Differentially Abundant Genera Between the Treatment Groups

**FIGURE 4:**
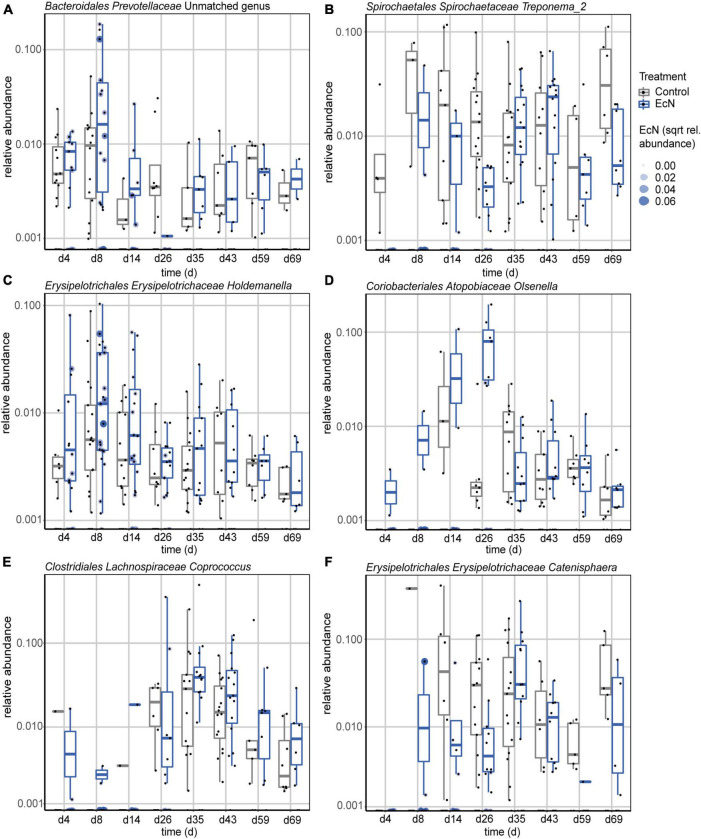
Relative abundances of differentially abundant genera in feces during the complete study period, including an unclassified genus in the family of *Prevotellaceae* **(A)**, *Treponema_2* **(B)**, *Holdemanella* **(C)**, *Olsenella* **(D)**, *Coprococcus* **(E)**, and *Catenisphaera* **(F)**. Shown genera resulted from comparing EcN-treated animals to control animals using a GAMLSS model. Probiotic intervention was used as the main variable for comparison and day of study and department were used as adjusting variables. Ear tag was used as identifier and “fdr” was chosen as the method for multiple testing adjustment (*p*.adjust < 0.05).

To investigate the effects of EcN on specific genera, the relative abundance of individual genera in feces were compared between the treatment groups. A GAMSLS model was used to identify significantly differently abundant genera. This resulted in the detection of six (out of 71) genera, including *Treponema_2*, *Holdemanella*, *Olsenella*, *Coprococcus*, *Catenisphaera*, and an unclassified genus in the family of *Prevotellaceae*, that were differentially abundant between the treatment groups (Figure 4). When only including pre- or post-weaning timepoints, six genera (out of 60) and two genera (out of 87) were found to be differentially abundant in fecal samples, respectively (Supplementary Figures 11, 12). Furthermore, a correlation was observed between the amount of EcN present in fecal samples, and the relative abundance of *Treponema_2*; samples that had a relative abundance of EcN higher than 0.05% did not contain the *Treponema_2* genus (Figure 4B). This relation was only observed during the pre-weaning period, since EcN was not detected in any of the post-weaning fecal samples. A complete overview of the presence of ASVs within the *Treponema_2* genus in feces from both treatment groups can be found in Supplementary Figure 13A. As it was previously demonstrated that ASVs within the genus of *Treponema* can be characterized up to species level using 16S rRNA amplicon sequencing (Hallmaier-Wacker et al., 2019), each ASV’s forward sequence was run through BLAST (see Text Footnote 3). This resulted in the detection of two ASVs with a 100% match with an earlier described invasive *Treponema* species (Mølbak et al., 2006), namely *Candidatus Treponema suis* (Supplementary Figure 13B). Taken together, these results show that EcN affected the relative abundance of specific genera in the gut, and that most of these changes were observed during the pre-weaning period.

### Intestinal Epithelial Gene Expression

**FIGURE 5:**
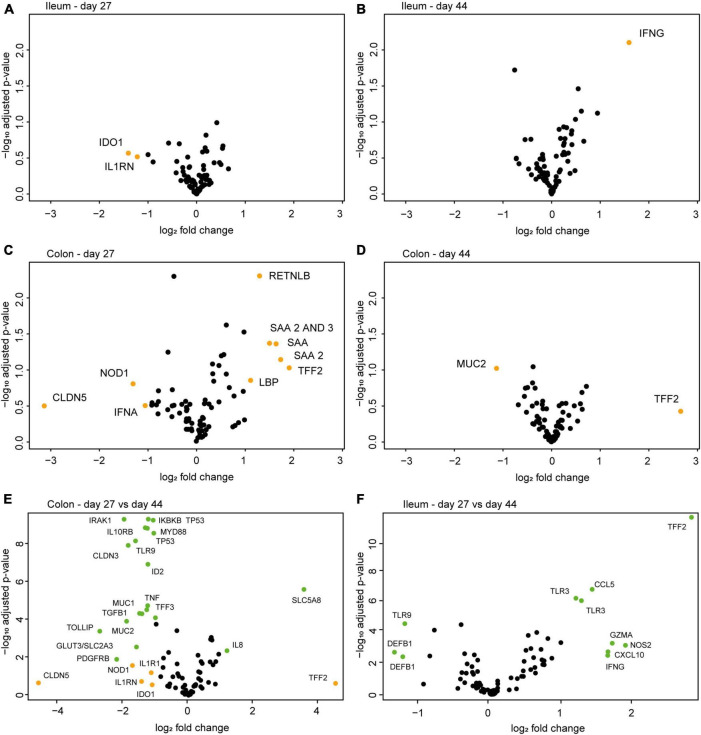
Volcano plots of differentially expressed genes between the control group and the EcN group (*n* = 8 per group) in mucosal scrapings of the ileum **(A,B)** and colon **(C,D)** on day 27 (pre-weaning) and day 44 (post-weaning) of the study. No significantly differentially expressed genes were observed between the treatment groups in any gut segment or time point. Fold change on the *x*-axis depicts the fold-change difference in the EcN treatment group compared to the control group. When comparing all samples from the colon **(E)** or ileum **(F)** from day 27 with those of day 44 (both treatment groups), several differentially expressed genes were identified. Fold change on the *x*-axis depicts the fold-change difference between time points. Orange points indicate the differentially expressed genes with a fold change > 2. Green points indicate differentially expressed genes with a fold change > 2 and a *p*-adj. < 0.05.

Gene expression analysis of mucosal scrapings revealed several differentially expressed (>2-fold) genes between the treatment groups (Figure 5, orange points). In the ileum, the genes IDO1 and IL1RN had a lower mean expression in EcN-treated animals on day 27, whereas IFNG showed a higher mean expression in the EcN group on day 44 (Figures 5A,B). As for the colon, a larger number of genes were differentially expressed between the treatment groups (Figures 5C,D). However, after correction for multiple testing, none of these genes were significantly differentially expressed. Comparison of data from day 27 (pre-weaning) with data from day 44 (post-weaning) demonstrated that twenty genes in the colon (Figure 5E) and 11 genes in the ileum (Figure 5F) were significantly differentially expressed (green points) between the time points.

### Effect of EcN Treatment on the Immune System

#### Vaccine Specific Antibody Responses

**FIGURE 6:**
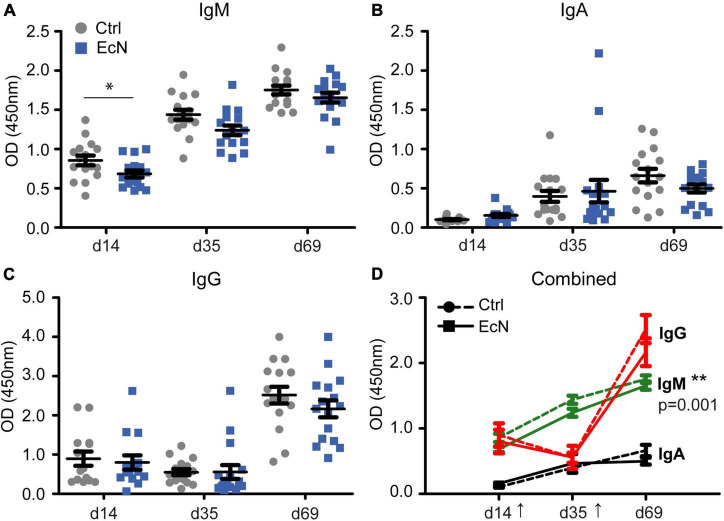
Serum levels of *Salmonella*-specific IgM, IgA, and IgG **(A–C)** prior to vaccination (day 14), 2 weeks after the initial vaccination (day 35), and 3 weeks after the booster vaccination (day 69). Plot **(D)** incorporates all results **(A–C)**, with the arrows indicating the time of vaccination (days 21 and 45). Every symbol represents a single animal. Two EcN-treated animals (squares; ■, solid lines) and two control animals (circles; •, dashed lines) were randomly selected from every pen, and followed over time (*n* = 16 per treatment group). Data are presented as the mean ± standard error of the mean (SEM). Asterisks are used to demonstrate significant differences between treatment groups after taking into account single time points (*p < 0.05 and **p < 0.01), and *p*-values indicate significant differences between treatment groups over time.

To assess the effect of EcN on the responsiveness of the immune system, a live-attenuated vaccine against *Salmonella enterica* serovar Typhimurium (Salmoporc^®^) was administered perorally on days 21 (primary vaccination) and 45 (booster vaccination) of the study. On days 14, 35, and 69 blood serum was collected and analyzed for vaccine-specific IgM, IgA, and IgG (Figures 6A–D). As expected, *Salmonella*-specific immunoglobulins increased significantly upon vaccination. *Salmonella*-specific IgM and IgA increased after both the primary and booster vaccination, whereas *Salmonella*-specific IgG increased only after the booster vaccination. Temporal analysis revealed that *Salmonella*-specific IgM in serum of EcN-treated was significantly lower than the control group (*p* = 0.001). A lower vaccine-specific IgM level could already be observed on day 14 of the study (*p* = 0.031), which was 1 week before the first vaccination with Salmoporc^®^. No significant differences between the EcN and control groups were found for *Salmonella*-specific IgA and IgG.

#### *Ex vivo* Stimulation of Peripheral Blood Mononuclear Cells and Mesenteric Lymph Node Cells

**FIGURE 7:**
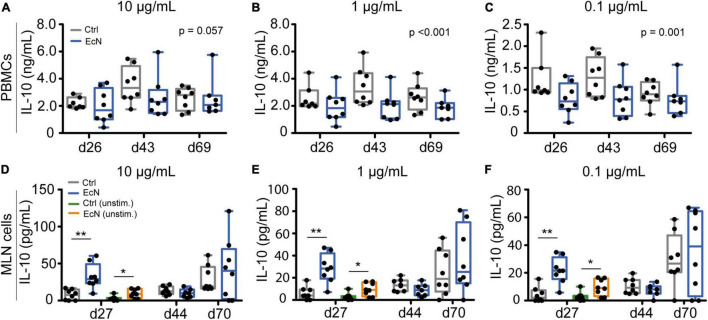
IL-10 production upon *ex vivo* stimulation of PBMCs and MLN cells. PBMCs **(A–C)** and MLN cells **(D–F)** were stimulated for 24 h with 10, 1, and 0.1 μg/mL of LPS, or left unstimulated (cell culture medium only). *P*-values indicate significant differences between treatment groups over time **(A–C)**, and asterisks are used to demonstrate significant differences between treatment groups after taking into account single time points (*p < 0.05 and **p < 0.01, **D–F**). Every point represents an individual animal from a separate pen (*n* = 7 or 8 per treatment group), and error bars represent standard deviations. Normal distribution and equal variances of data were checked and log-transformed when necessary. No differences in IL-10 production by unstimulated MLN cells were observed post-weaning and are therefore not presented.

To assess whether EcN modulated TNFα and IL-10 secretion, different concentrations of LPS and ConA were used for *ex vivo* stimulation of isolated immune cells. Temporal analysis (using a LMM) illustrated that EcN did not affect TNFα production by stimulated PBMCs and MLN cells (data not shown). In contrast, PBMCs from EcN-treated animals produced a lower amount of IL-10 upon stimulation with LPS in comparison to the control group (Figures 7A–C). This result was observed after stimulation with 10, 1, as well as 0.1 μg/mL LPS (*p* = 0.057, *p* = <0.001 and *p* = 0.001, respectively). PBMCs stimulated with ConA also produced a lower amount of IL-10 (Supplementary Figure 14). There were no significant differences in IL-10 production by stimulated MLN cells. However, LPS stimulated MLN cells from EcN-treated animals produced significantly higher amounts of IL-10 when only taking into account the pre-weaning time point (day 27) (Figures 7D–F). The same trend was observed after stimulation with 5 μg/mL of ConA (*p* = 0.07, Supplementary Figure 14). Interestingly, unstimulated MLN cells from EcN-treated animals also produced higher levels of IL-10 in comparison to unstimulated MLN cells from control animals (*p* = 0.016), and again this was only observed pre-weaning.

#### Innate and Adaptive Immune Cell Analysis

**FIGURE 8:**
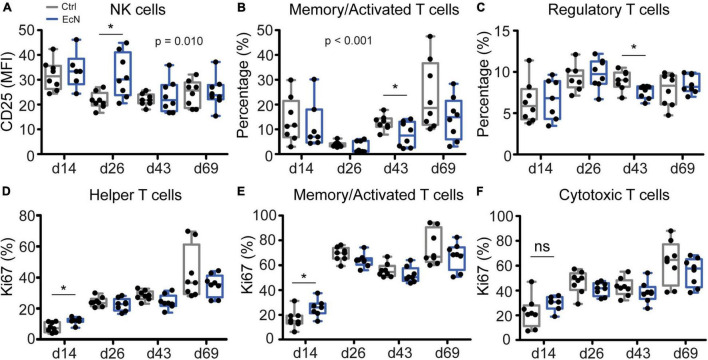
Analysis of innate and adaptive immune cells in PBMCs. *P*-values indicate significant differences between treatment groups over time **(A,B)**, and asterisks are used to demonstrate significant differences between treatment groups after taking into account single time points (*p < 0.05, **C–E**). Data were presented as the mean fluorescent intensity (MFI; **A**), percentage of cells (%; **B,C**), or percentage of proliferating cells (Ki67%; **D–F**). Every point represents an individual animal from a separate pen (*n* = 7 or 8 per treatment group), and error bars represent standard deviations. Normal distribution and equal variances of data were checked and log-transformed when necessary.

To determine whether there was an effect of EcN on specific innate and adaptive immune cell populations, cell analysis was performed on isolated PBMCs (Supplementary Table 1) and MLN cells (Supplementary Table 2) from a subset of animals. The number of DCs (pDC, cDC1, and cDC2) or DC maturation status (determined by CD80/86 upregulation) was not significantly different between the EcN and control groups. In addition, no differences between the control and treatment group were observed for other immune cells from the MLN (Supplementary Table 2). Temporal analysis of immune cells from PBMCs revealed that EcN significantly (*p* = 0.010) enhanced expression of the activation marker CD25 on NK cells (CD3^–^CD8α^+^), which was most evident on day 26 pre-weaning (*p* = 0.015, Figure 8A). However, EcN treatment did not significantly increase NK cell numbers in PBMCs (Supplementary Table 1). Furthermore, temporal analysis of specific T cell populations showed that the percentage of memory/activated T cells (CD3^+^TCRγδ^–^CD4^+^CD8α^+^) was significantly lower (*p* = <0.001) in the EcN treatment group (Figure 8B). Moreover, analysis of single time points showed a significant reduction of memory/activated T cells on day 44 (*p* = 0.042). Similarly, a significant reduction of regulatory T cells (CD3^+^TCRγδ^–^CD4^+^CD25*^high^*Foxp3^+^) was observed on day 44 of the study (*p* = 0.016, Figure 8C). Next to cell number, maturation and activation, proliferative responses of different T cell populations were investigated by using the proliferation marker Ki67. EcN-treated animals had a significantly higher percentage of Ki67-positive T helper cells (CD3^+^TCRγδ^–^CD4^+^) and memory/activated T cells (CD3^+^TCRγδ^–^CD4^+^CD8α^+^) on day 14 of the study (*p* = 0.011 and *p* = 0.030, respectively) (Figures 8D,E). Albeit not significant, a similar pattern could be observed for CD3^+^TCRγδ^+^ CD8α^+^Ki67^+^ T cells (cytotoxic T cells, Figure 8F).

## Discussion

For over 100 years EcN has been extensively studied *in vitro* or in animal models to understand its probiotic properties, but to date, no studies have investigated its effects on the porcine gut microbiota and the immune system when administered in early life. It is well known that the conditions (e.g., diet and housing) in the neonatal phase and during early life shape the development of the GIT and the immune system (Zmora et al., 2019). Therefore, we hypothesized that by administration of EcN in the immediate postnatal period leads to colonization of the intestine and competition with overt pathogens and pathobionts that often increase in abundance due to stressors in the post-weaning period. In addition, we hypothesized that gut-adherent EcN would interact with the gut epithelial barrier and the innate and adaptive arms of the immune system.

The EcN was detected in fecal samples for at least 4 weeks (primarily pre-weaning) after administration of EcN every other day from day 2 until day 44. EcN reached highest abundance in feces on days 8 and 14 but was not detected after weaning. This may be due to the increasing complexity of the gut microbiota over time, and the dramatic shift of the gut microbiota as a result of weaning (due to changes in diet and housing). These factors may hinder growth, subsequent colonization and detection of EcN in the GIT. However, in three piglets EcN was detected in jejunal or ileal digesta at the end of the study (day 70), demonstrating that in some pigs EcN can persist in the GIT. This is consistent with other studies that showed that EcN could be detected in feces of post-weaning pigs after oral administration (Duncker et al., 2006), and in pigs that were not deliberately treated with EcN (Kleta et al., 2006). In another study where EcN was administered to 3-month-old pigs for seven consecutive days, EcN was detected in feces approximately 4 weeks after the last administration (Barth et al., 2009). This may be due to the larger number of EcN administered (i.e., 3.75–37.5 × 10^8^ CFU EcN per kg of body weight per day) or the use of a different detection method. Another study administering EcN (3.5 × 10^10^ CFU) for 14 consecutive days to 4–5 months old pigs, reported that EcN was not recovered from intestinal contents using cultivation-dependent methods (Šmajs et al., 2012). Collectively these studies indicate that EcN can persist in the intestine during the pre-weaning period, but is not efficiently colonizing the porcine GIT. This is consistent with studies on EcN colonization in humans (Wassenaar, 2016), and can also be explained by the fact that facultative anaerobic bacterial species typically represent only 0.1% of the bacteria that are present in the anaerobic environment of the colon (Eckburg et al., 2005). Colonization of EcN in an established intestinal microbiome may also be more challenging, which could explain why EcN administration does not lead to colonization or persistence post-weaning.

Since EcN was primarily present pre-weaning, it was considered likely that EcN exerted effects on the gut microbiota composition during this period. We found that EcN reduced the diversity within the phylum of Proteobacteria in the porcine GIT during the pre-weaning period, as shown in another study (Šmajs et al., 2012). These results are in line with the antagonistic effects of EcN on growth of closely related taxa in the family of *Enterobacteriaceae* (Lodinová-Žádníková and Sonnenborn, 1997; Jacobi and Malfertheiner, 2011; Sassone-Corsi et al., 2016; Sonnenborn, 2016). Moreover, our results show that presence of EcN altered the relative abundance of specific genera, with a particularly interesting correlation for the genus *Treponema_2*; relative presence of EcN higher than 0.05% in fecal samples corresponded with absence of the *Treponema_2* genus. This is an interesting finding, given *Treponema* is often associated with pathogenic species (Mølbak et al., 2006; Hallmaier-Wacker et al., 2019; Giffin et al., 2020). The antagonistic effect against the *Treponema* genus is in line with observed effects from other known probiotics such as *Bifidobacterium* species (Belkacemi et al., 2020), *Lactobacillus fermentum* and *Pediococcus acidilactici* (Wang et al., 2019). The genus *Treponema* contains a number of pathogenic and symbiotic bacteria that can be found in vastly different anatomical and environmental habitats (Hallmaier-Wacker et al., 2019). In 2006, the 16S rRNA gene of a *Treponema* strain was PCR amplified after isolation in pig host tissue. This strain, also known as *Candidatus Treponema suis*, was found to be associated with colitis considering its invasion of the surface epithelium as well as superficial parts of the mucosa (Mølbak et al., 2006). Two of the *Treponema_2* ASVs within our dataset were identical in sequence to the 16S rRNA gene of this strain. When plotting these two ASVs over time, a very interesting temporal pattern could be discerned in both treatment groups (Supplementary Figure 13B), with higher abundances observed in the immediate post-weaning period up to 2 weeks post-weaning (days 35 and 43, respectively). As piglets are known to suffer from post-weaning diarrhea especially in the first week post-weaning up to the second-week post-weaning, the potential role of *Candidatus Treponema suis* in post-weaning diarrhea warrants further investigation. Although *Treponema* and Proteobacteria include harmless commensal bacterial species, they are primarily known as potential pathogens. Thus, the association between EcN and reduced Proteobacteria diversity (pre-weaning) as well as the potential antagonistic effect against *Treponema* suggest a beneficial effect on the porcine gut microbiota composition.

In contrast to the little information available regarding the effects of EcN on the gut microbiota, more is known about the bacterial–epithelial crosstalk between EcN and the intestinal epithelial cells (Sonnenborn and Schulze, 2009; Wassenaar, 2016). In mice with inflammatory colitis, EcN strengthened the epithelial barrier by enhancing expression of tight junction proteins ZO-1 (Ukena et al., 2007). In addition, EcN altered the expression and distribution of ZO-2 protein in *in vitro* cultured human epithelial cells (Zyrek et al., 2007). Differential expression of these zonulin genes was not observed in our study, possibly due to the use of conventionally reared healthy animals. Significant differences in gene expression were observed between days 27 and 44, particularly in the colon. Albeit not significant, our data also showed that on day 27 nine genes were differentially expressed (at least twofold) in the colon of EcN-treated piglets, while on day 44 this was the case for only two genes. This could imply that EcN is more likely to induce changes in the gut epithelial barrier in the pre-weaning period.

Supplementation of EcN in early life aims at providing the gut with advantageous commensal bacteria that directly or indirectly (by affecting the gut microbiota composition or gut barrier function) influence the immune responsiveness. Considering the decrease of Proteobacteria diversity, EcN may have hindered pathogenic enterobacteria, thereby influencing the immune response. As for direct effects, several studies indicated that EcN activates Toll-like Receptor 4 (TLR-4) that recognizes the lipid A component of semi-rough type LPS present in the outer cell membrane of EcN (Grozdanov et al., 2002; Grabig et al., 2006; Adam et al., 2010), and has been identified along the porcine GIT and on porcine blood and lymphoid DCs (Gourbeyre et al., 2015; Auray et al., 2016; Shao et al., 2016). Conversely, TLR-4 expression in the ileum, colon or MLN of 1-week-old gnotobiotic piglets was not altered by EcN (Splichal et al., 2019). We measured no significant effects of EcN on genes involved in gut barrier function, or on DC maturation (upregulation of CD80/86). In our previous *in vitro* study, we showed an EcN concentration-dependent increase of the DC maturation marker CD80/86 upon stimulation with EcN (Geervliet et al., 2020), but in that study, DCs were in direct contact with the bacteria. Interestingly, we did detect a significant increase of the activation marker CD25 on NK cells in blood, which was most evident on day 26 of the study (pre-weaning). Activated NK cells produce a variety of inflammatory cytokines (e.g., IFN-γ), followed by cytokine-induced downstream signaling cascades that contribute to their immune response against infection (Abel et al., 2018). Previous studies have demonstrated that EcN is able to enhance the frequency and function of NK cells in systemic tissues and blood from gnotobiotic pigs after a virulent human rotavirus challenge (Vlasova et al., 2016; Michael et al., 2021a). Moreover, EcN biofilm treatment also showed to enhance NK cell activity in blood mononuclear cells from malnourished piglets transplanted with human infant fecal microbiota (Michael et al., 2021b). Our results demonstrate that EcN altered the immune responsiveness in the pre-weaning period, but further (challenge) studies are required to determine if enhanced NK cell responses induced by EcN indeed provide protection against infectious agents.

EcN did not only modulate innate immune responses, but also affected adaptive immune responses, the latter being the basis for effective immunization against infections. For example, a modest but significantly enhanced proliferation of T helper cells and Memory/Activated T cells in PBMCs was observed on day 14 of the study. On the other hand, EcN reduced the number of Memory/Activated T cells and regulatory T cells in blood, and enhanced IL-10 production by LPS stimulated MLN cells. These results suggest that EcN induced a more regulatory or anti-inflammatory state of the local gut-associated immune system. In contrast, temporal analysis revealed that LPS stimulated PBMCs from EcN-treated animals produced lower levels of IL-10, indicating different effects of EcN on the local (MLN) and systemic (PBMC) immune system. These differences could be explained by the fact that the MLN is the main site for tolerance induction, and a large number of cells (e.g., DCs) that reside in the MLN are still immature or naive. Moreover, the reduction of *Salmonella*-specific IgM in EcN-treated animals may also imply immune tolerance. However, a significant reduction of *Salmonella*-specific IgM was already observed prior to vaccination (day 14), which could mean that *Salmonella*-specific antibodies were already present in the pigs’ blood or that antibodies to other *Enterobacteriaceae* cross-react with components of the ELISA. Our observation that EcN primarily induced changes pre-weaning supports our hypothesis that repeated administration of EcN in early life affects the porcine gut microbiota and the developing innate and adaptive arms of the porcine immune system. Our results also illustrated that EcN did not consistently persist during the post-weaning period, and that EcN did not affect the gut barrier function or the vaccination response. Compared to the pre-weaning period, effects observed post-weaning were modest. EcN’s ability to reduce the diversity of taxa within the phylum of Proteobacteria, its seemingly antagonistic effect against *Treponema*, and its effect on cells (e.g., NK cell activation) and cell responses (e.g., IL-10 production by MLN cells and PBMCs) imply beneficial effects, which should be further investigated in future infection studies. Taken together, the results of this study support the concept that the pre-weaning period provides a ‘window of opportunity’ to modulate the gut microbiota and the immune system through dietary interventions such as EcN.

## Data Availability Statement

The data presented in the study are deposited in the 4TU.ResearchData repository, under the following doi: https://doi.org/10.4121/15060177.v1.

## Ethics Statement

The animal study was reviewed and approved by Dutch Central Authority for Scientific Procedures on Animals (CCD).

## Author Contributions

MG, HFJS, HS, ET, HV, and JW: conceptualization. MG, HV, GA, CW, and KS: data curation. MG, HV, CJ, KS, HFJS, HS, ET, and JW: methodology. MG and HV: software, validation, formal analysis, writing—original draft preparation, writing and editing, and visualization. MG, GA, ET, and HV: investigation. HH, MG, HFJS, HS, and HV: resources. CJ, VR, HFJS, HS, ET, and JW: supervision. HFJS, HS, and JW: project administration and funding acquisition. All authors contributed to manuscript revision, read, and approved the submitted version.

## Conflict of Interest

The authors declare that the research was conducted in the absence of any commercial or financial relationships that could be construed as a potential conflict of interest.

## Publisher’s Note

All claims expressed in this article are solely those of the authors and do not necessarily represent those of their affiliated organizations, or those of the publisher, the editors and the reviewers. Any product that may be evaluated in this article, or claim that may be made by its manufacturer, is not guaranteed or endorsed by the publisher.

## Footnotes

1
https://www.ensembl.org/index.html

2
https://bioinfo.ut.ee/primer3-0.4.0/

3
https://blast.ncbi.nlm.nih.gov/Blast.cgi
