# Supplementary material for: Effects of Escherichia coli Nissle 1917 on the Porcine Gut Microbiota, Intestinal Epithelium and Immune System in Early Life
Source: Front Microbiol. 2022 Feb 25;13:842437. doi: 10.3389/fmicb.2022.842437 (PMC8914288; doi:10.3389/fmicb.2022.842437)
Supplement: Supplementary file 3 [file Data_Sheet_3.DOCX]

Supplementary Material

Supplementary Material belonging to the Original Research Article entitled: Effects of *E. coli* Nissle 1917 on the Porcine Gut Microbiota, Intestinal Epithelium and Immune System in Early Life. All supplementary files are deposited under DOI: 10.4121/15060177.

**Table S1.** Analysis of innate and adaptive immune cells in PBMCs between treatment groups over time.

| Subset | Phenotype | Unpaired T-test | | | | Linear Mixed Model |
| --- | --- | --- | --- | --- | --- | --- |
|  |  | D14 | D26 | D43 | D69 |  |
|  |  | p-value [CI] | p-value [CI] | p-value [CI] | p-value [CI] | |
| pDCs (%) | CD14^−^CD4^+^CD172a^+^CADM1^-^ | ns | ns | ns | ns | T (<0.001) |
| pDCs (mat.) | CD14^−^CD4^+^CD172a^+^CADM1^-^CTLA4-Ig^+^ | ns | ns | ns | ns | T (<0.001) |
| cDC1 (%) | CD14^−^CD4^−^CD172a^low^CADM1^+^ | ns | ns | ns | ns | T (<0.001) |
| cDC1 (mat.) | CD14^−^CD4^−^CD172a^low^CADM1^+^ CTLA4-Ig^+^ | ns | ns | ns | ns | T (0.018) |
| cDC2 (%) | CD14^−^CD4^−^CD172a^+^CADM1^+^ | ns | ns | ns | ns | T (0.035) |
| cDC2 (mat.) | CD14^−^CD4^−^CD172a^+^CADM1^+^ CTLA4-Ig^+^ | ns | ns | ns | ns | T (<0.001) |
|  | | | | | | |
| NK cells (%) | CD3^-^CD8α^+^ | ns | ns | ns | ns | T (0.051) |
| NK cells (act.) | CD3^-^CD8α^+^CD25^+^ | ns | **0.015↑[-17.7 to -2.45]** | ns | ns | **G (0.010)**, T (<0.001) |
| NK cells (Ki67%) | CD3^-^CD8α^+^Ki67^+^ | ns | ns | ns | ns | T (<0.001) |
| γδ T cells (%) | CD3^+^TCRγδ^+^ | ns | ns | ns | ns | T (<0.001) |
| γδ T cells (Ki67%) | CD3^+^TCRγδ^+^Ki67^+^ | ns | ns | ns | ns | T (<0.001) |
| CTLs (%) | CD3^+^TCRγδ^-^CD8α^+^ | ns | ns | ns | ns | T (<0.001) |
| CTLs (Ki67%) | CD3^+^TCRγδ^-^CD8α^+^Ki67^+^ | ns | ns | ns | ns | T (<0.001) |
| T helper (%) | CD3^+^TCRγδ^-^CD4^+^ | ns | ns | ns | ns | T (<0.001) |
| T helper (Ki67%) | CD3^+^TCRγδ^-^CD4^+^Ki67^+^ | **0.011↑ [-7.11 to -1.11]** | ns | ns | ns | T (<0.001) |
| Mem./Act. (%) | CD3^+^TCRγδ^-^CD4^+^CD8α^+^ | ns | ns | **0.042↓[0.014 to 0.587]** | ns | **G (<0.001)**, T (<0.001) |
| Mem./Act. (Ki67%) | CD3^+^TCRγδ^-^CD4^+^CD8α^+^ki67^+^ | **0.030↑ [-17.1 to -1.01]** | ns | ns | ns | T (<0.001), I() |
| Tregs (%) | CD3^+^TCRγδ^-^CD4^+^CD25^high^Foxp3^+^ | ns | ns | **0.016↓ [0.31 to 2.47]** | ns | T (<0.001) |
| Tregs (Ki67%) | CD3^+^TCRγδ^-^CD4^+^CD25^high^Foxp3^+^Ki67^+^ | ns | ns | ns | ns | T (<0.001) |

An Unpaired Student’s T-tests and a Linear Mixed Model (LMM) were performed to analyse differences between treatment groups per time point and over time, respectively (n = 7 or 8 per treatment group). Abbreviations: G; group effect, T; time effect, I; group-time interaction. Ns; not significant, p <0.05; significant, 0.05 > p <0.1; trend. Arrows indicate if effects (e.g., percentage of cells) in the treatment group are higher (↑) or lower (↓ ) compared to the control group. For all results that are significant or show a trend, the 95% confidence intervals (CI) on the difference between the means are given.

| Subset | Phenotype | Unpaired T-test Linear Mixed Model | | | |
| --- | --- | --- | --- | --- | --- |
|  |  | D27 | D44 | D70 |  |
|  |  | p-value [CI] | p-value [CI] | p-value [CI] | |
| pDCs (%) | CD14^−^CD4^+^CD172a^+^CADM1^-^ | ns | ns | 0.064**↓** [-0.021 to 0.659] | T (<0.001) |
| pDCs (mat.) | CD14^−^CD4^+^CD172a^+^CADM1^-^CTLA4-Ig^+^ | ns | ns | 0.092**↓** [-6.092 to 72.39] | T (<0.001) |
| cDC1 (%) | CD14^−^CD4^−^CD172a^low^CADM1^+^ | ns | ns | ns | T (<0.001) |
| cDC1 (mat.) | CD14^−^CD4^−^CD172a^low^CADM1^+^CTLA4-Ig^+^ | ns | ns | ns | T (<0.001) |
| cDC2 (%) | CD14^−^CD4^−^CD172a^+^CADM1^+^ | ns | ns | ns | T (<0.001) |
| cDC2 (mat.) | CD14^−^CD4^−^CD172a^+^CADM1^+^CTLA4-Ig^+^ | ns | ns | ns | ns |
|  | | | | | |
| NK cells (%) | CD3^-^CD8a^+^ | n/a | n/a | n/a | n/a |
| NK cells (act.) | CD3^-^CD8α^+^CD25^+^ | n/a | n/a | n/a | n/a |
| NK cells (Ki67%) | CD3^-^CD8α^+^Ki67^+^ | n/a | n/a | n/a | n/a |
| γδ T cells (%) | CD3^+^TCRγδ^+^ | ns | ns | ns | T (<0.001) |
| γδ T cells (Ki67%) | CD3^+^TCRγδ^+^Ki67^+^ | ns | 0.093↓ [-0.643 to 7.300] | ns | T (0.022) |
| CTLs (%) | CD3^+^TCRγδ^-^CD8α^+^ | ns | 0.091↑ [-6.011 to 0.5034] | ns | T (0.004) |
| CTLs (Ki67%) | CD3^+^TCRγδ^-^CD8α^+^Ki67^+^ | ns | ns | ns | T (<0.001) |
| T helper (%) | CD3^+^TCRγδ^-^CD4^+^ | ns | ns | ns | T (<0.001) |
| T helper (Ki67%) | CD3^+^TCRγδ^-^CD4^+^Ki67^+^ | ns | ns | ns | T (0.006) |
| Mem./Act. (%) | CD3^+^CD4^+^CD8α^+^ | ns | ns | ns | T (<0.001) |
|  | CD3^+^CD4^high^CD8α^low^ | ns | ns | ns | T (<0.001) |
|  | CD3^+^CD4^high^ CD8α^low^ki67^+^ | ns | ns | ns | T (0.002) |
|  | CD3^+^CD4^low^CD8α^high^ | ns | ns | ns | T (0.038) |
|  | CD3^+^CD4^low^ CD8α^high^ki67^+^ | ns | ns | ns | T (<0.001) |
| Tregs (%) | CD3^+^TCRγδ^-^CD4^+^CD25^high^Foxp3^+^ | ns | ns | ns | T (<0.001) |
| Tregs (Ki67%) | CD3^+^TCRγδ^-^CD4^+^CD25^high^Foxp3^+^Ki67^+^ | n/a | n/a | n/a | n/a |

**Table S2.** Analysis of innate and adaptive immune cells in MLN cells between treatment groups over time.

An Unpaired Student’s T-tests and a Linear Mixed Model (LMM) were performed to analyse differences between treatment groups per time point and over time, respectively (n = 7 or 8 per treatment group). Abbreviations: G; group effect, T; time effect, I; group-time interaction. Ns; not significant, p <0.05; significant, 0.05 > p <0.1; trend. Arrows indicate if effects (e.g., percentage of cells) in the treatment group are higher (↑) or lower (↓ ) compared to the control group. For all results that are significant or show a trend, the 95% confidence intervals (CI) on the difference between the means are given.


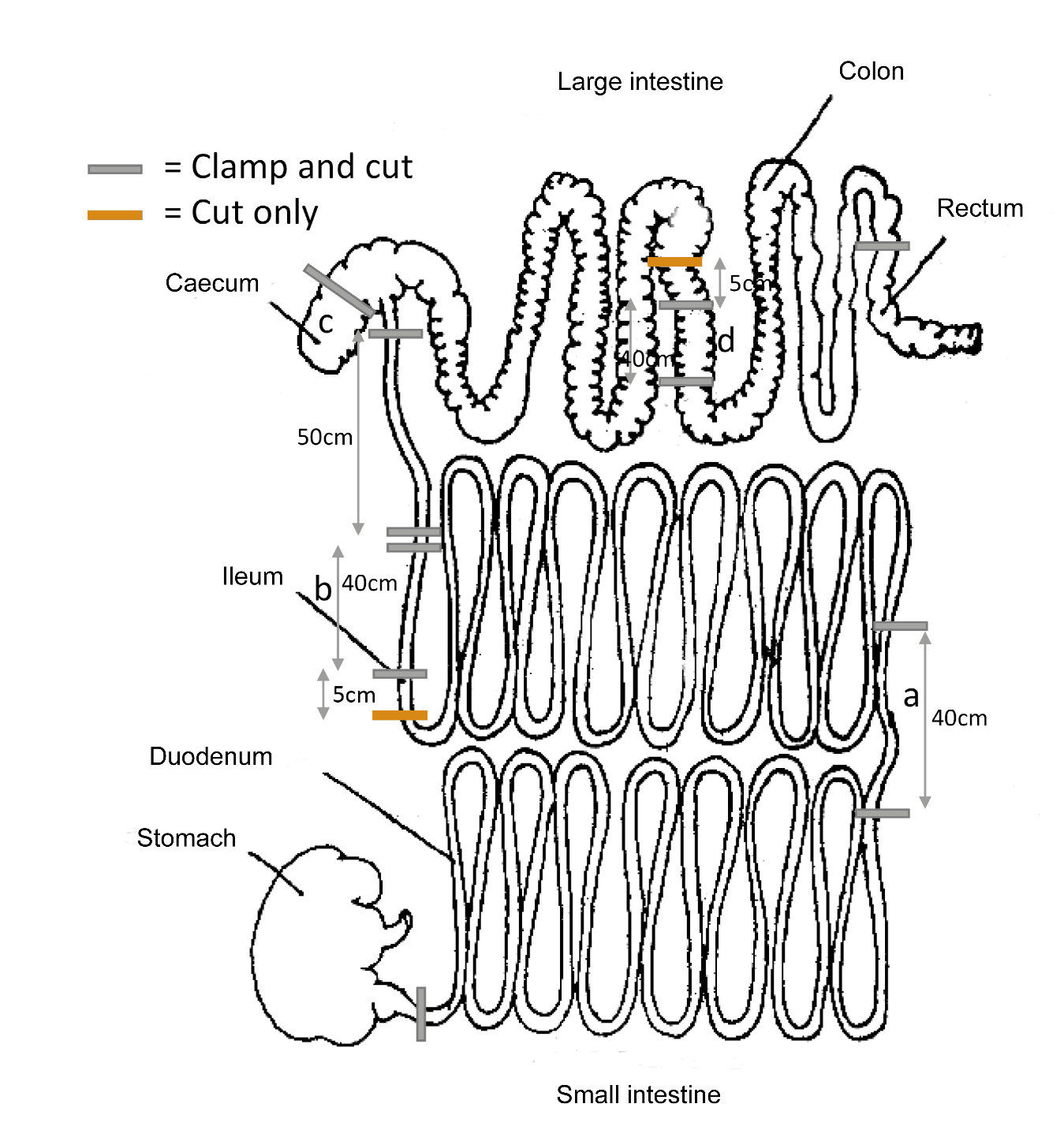


**Figure S1.** Visual representation of the standardized division of GIT segments at dissection. The jejunum segment (a) was taken by clamping off a 40 cm piece halfway the small intestine after which a cut was made on both sides in order to place the 40 cm piece in a separate container. The ileum segment (b) was taken by placing clamps at 50 cm and 90 cm proximal to the ileocaecal valve. Caecum luminal content (c) was taken by first isolating the caecum content using a clamp after which a cut was made in order to place the caecum in a separate container for further processing. Halfway the colon, a 40 cm segment (clamped off) was taken distally from mid-point. For both ileum and colon, a 5 cm segment was taken proximally to the 40 cm segment that was to be used for transcriptomics.


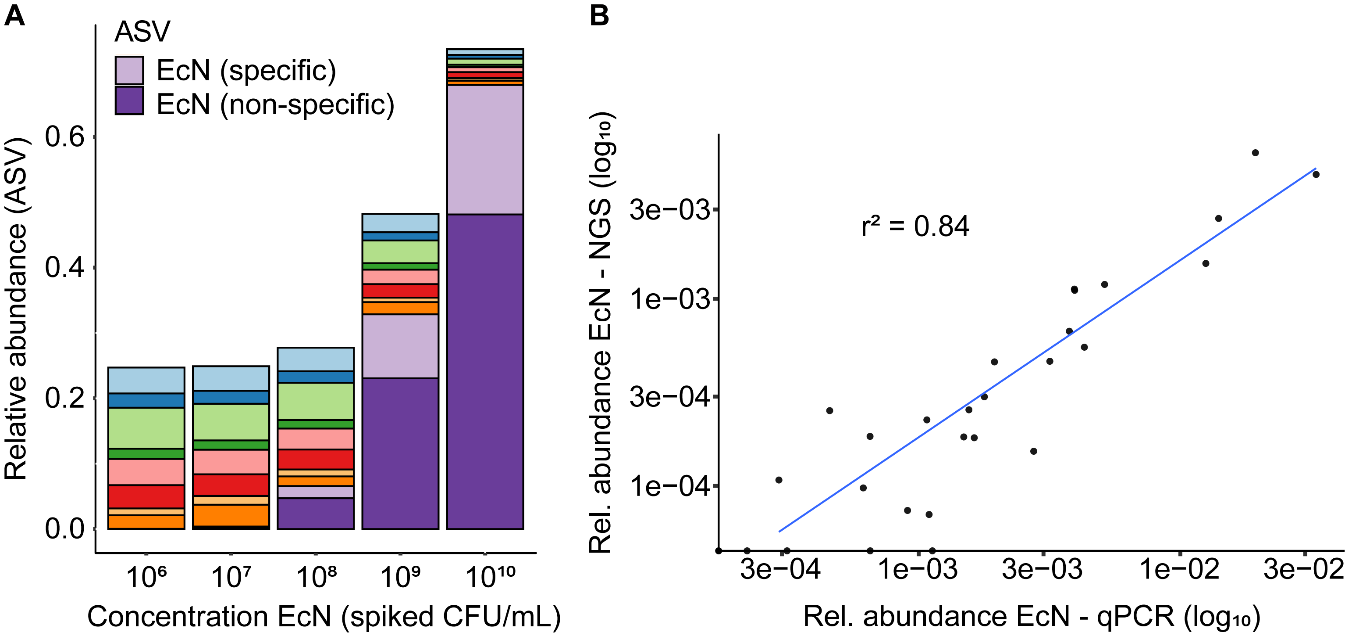


**Figure S2.** (**A**) Bar plot of top 10 abundant ASVs in the spiked samples. The abundance of two 16S rRNA gene sequences (ASVs), both of which are present in the EcN genome, increased in proportion to the amounts of EcN added to the spiked samples. One of these two ASVs was found to be specific to EcN in the NGS dataset and was therefore used to identify EcN in faeces. It should be noted, however, that by including solely the EcN-specific ASV, the relative abundance of EcN is underestimated by a two-to-three fold. (**B**) Validation of EcN in NGS dataset using qPCR. Relative abundance of EcN calculated using the specific EcN qPCR compared to the relative abundance of EcN calculated using the EcN-specific ASV in the NGS dataset. From the regression line (blue), the correlation determinant r^2^ = 0.84, indicates a high correlation of the two methods for EcN quantification in faecal samples. Additionally, with the use of a CT value of 33 as threshold with qPCR, each positive outcome of EcN in the NGS dataset was validated.


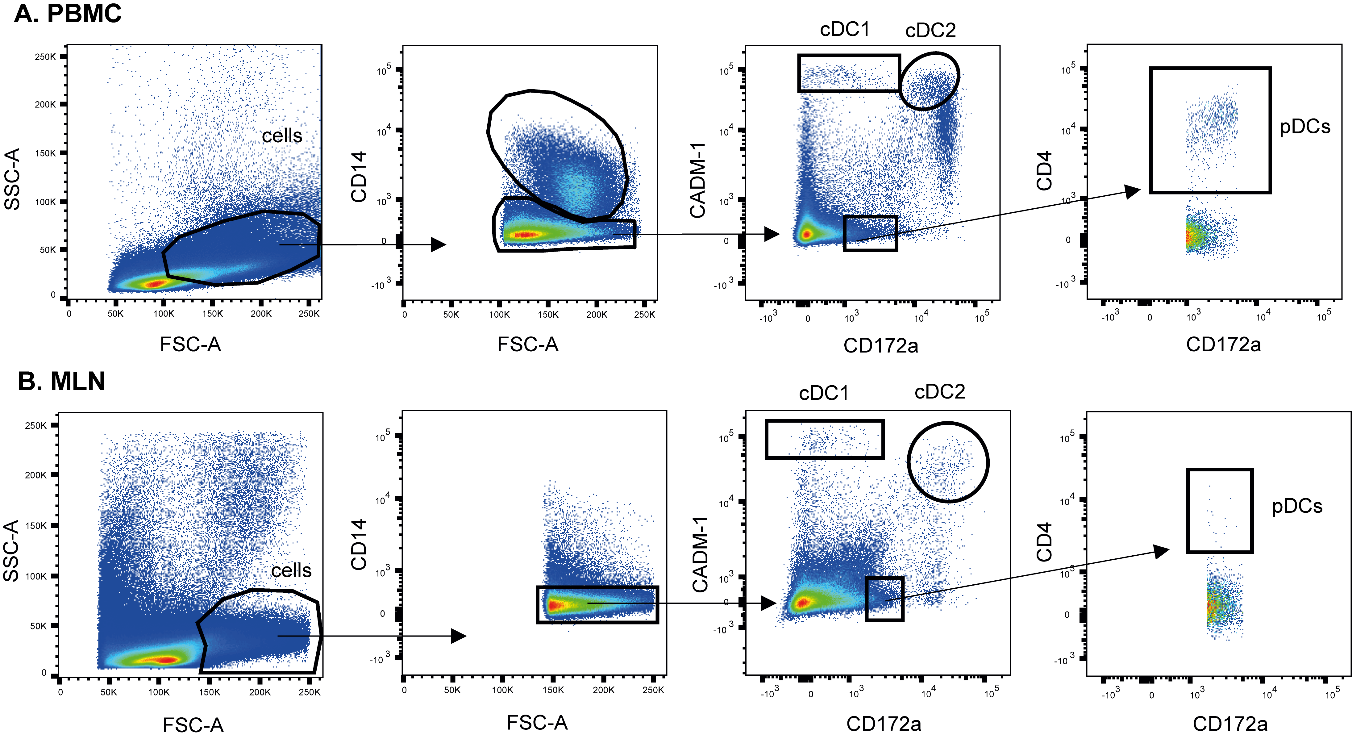


**Figure S3.** Gating strategy for the identification of DC subsets in porcine PBMCs and MLN cells. After doublet exclusion and discrimination of viable (live) and non-viable (death) cells, live cells were gated based on scatter light (forward scatter; FSC, and side scatter; SSC). Antibodies that specifically recognize CD14, CD172a, CADM1 and CD4 in were used to identify DC subsets in PBMCs (**A**) and MLN cells (**B**).


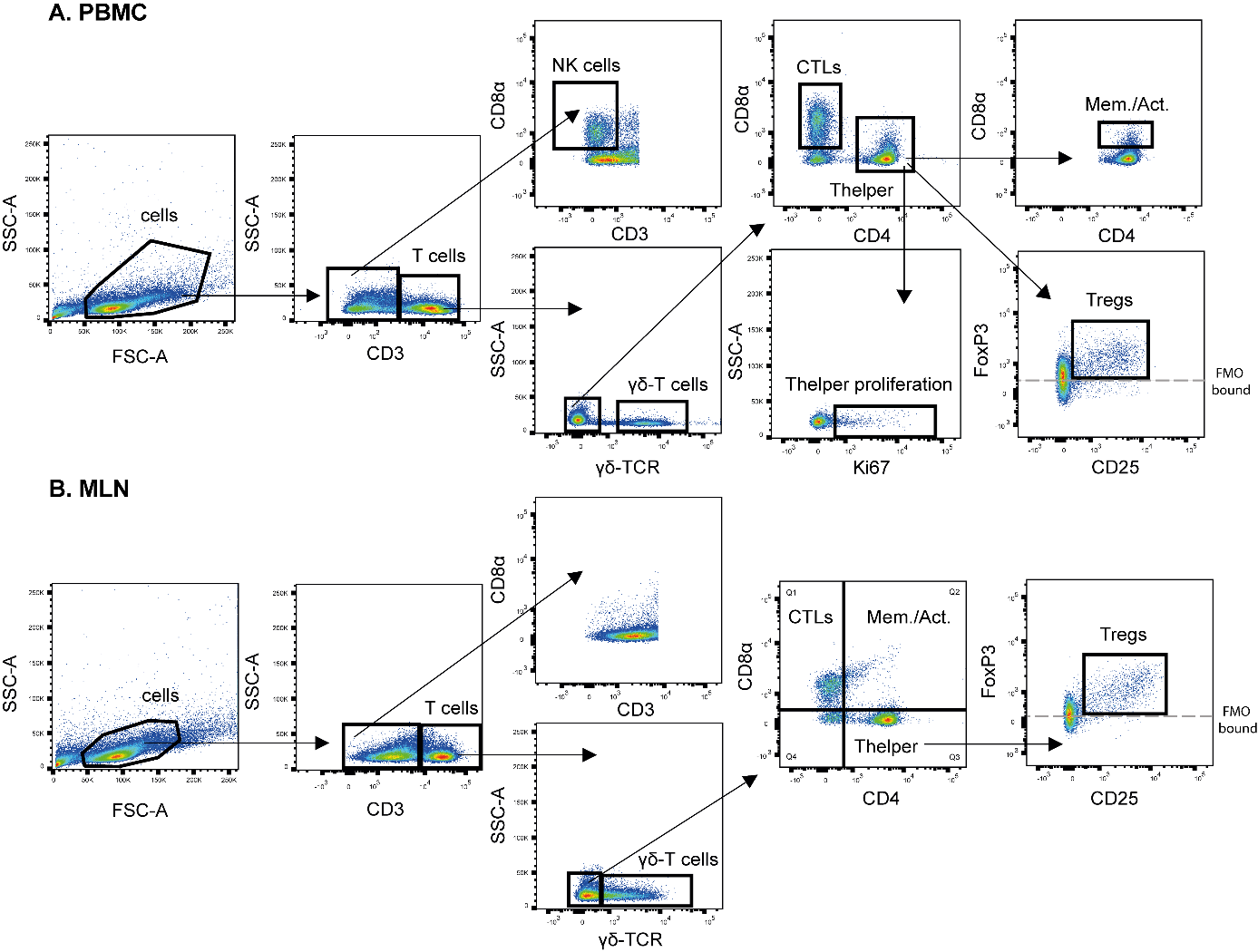


**Figure S4.** Gating strategy for the identification of NK cells and T cell subsets in porcine PBMCs and MLN cells. After doublet exclusion and discrimination of viable (live) and non-viable (death) cells, live cells were gated based on scatter light (forward scatter; FSC, and side scatter; SSC). Antibodies that specifically recognize CD3, CD8α, TCRγδ, CD4, FoxP3 and CD25 were used to identify NK cells and T cell subsets in PBMCs (A) and MLN cells (B). Ki67 was used to determine cell proliferation. The gating strategy for determining T helper cell proliferation is presented as an example (A).


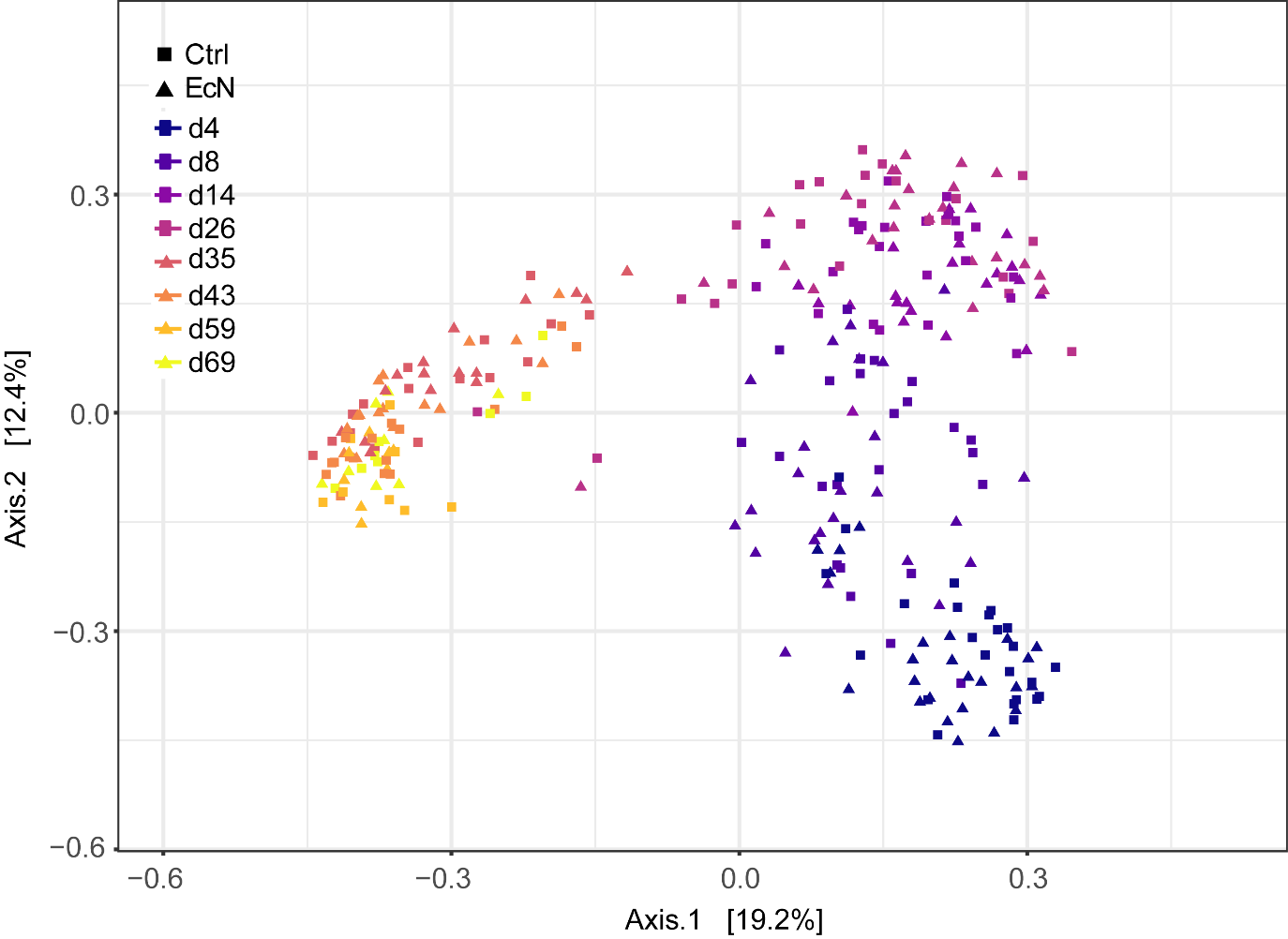


**Figure S5.** Principal Coordinates Analysis (PCoA) of beta-diversity values (Bray-Curtis dissimilarities) in faecal samples over time (d4-69). Every point represents a faecal sample from an individual animal, and colors correspond to time points. Control and EcN-treated animals are represented by squares (■) and triangles (▲), respectively.


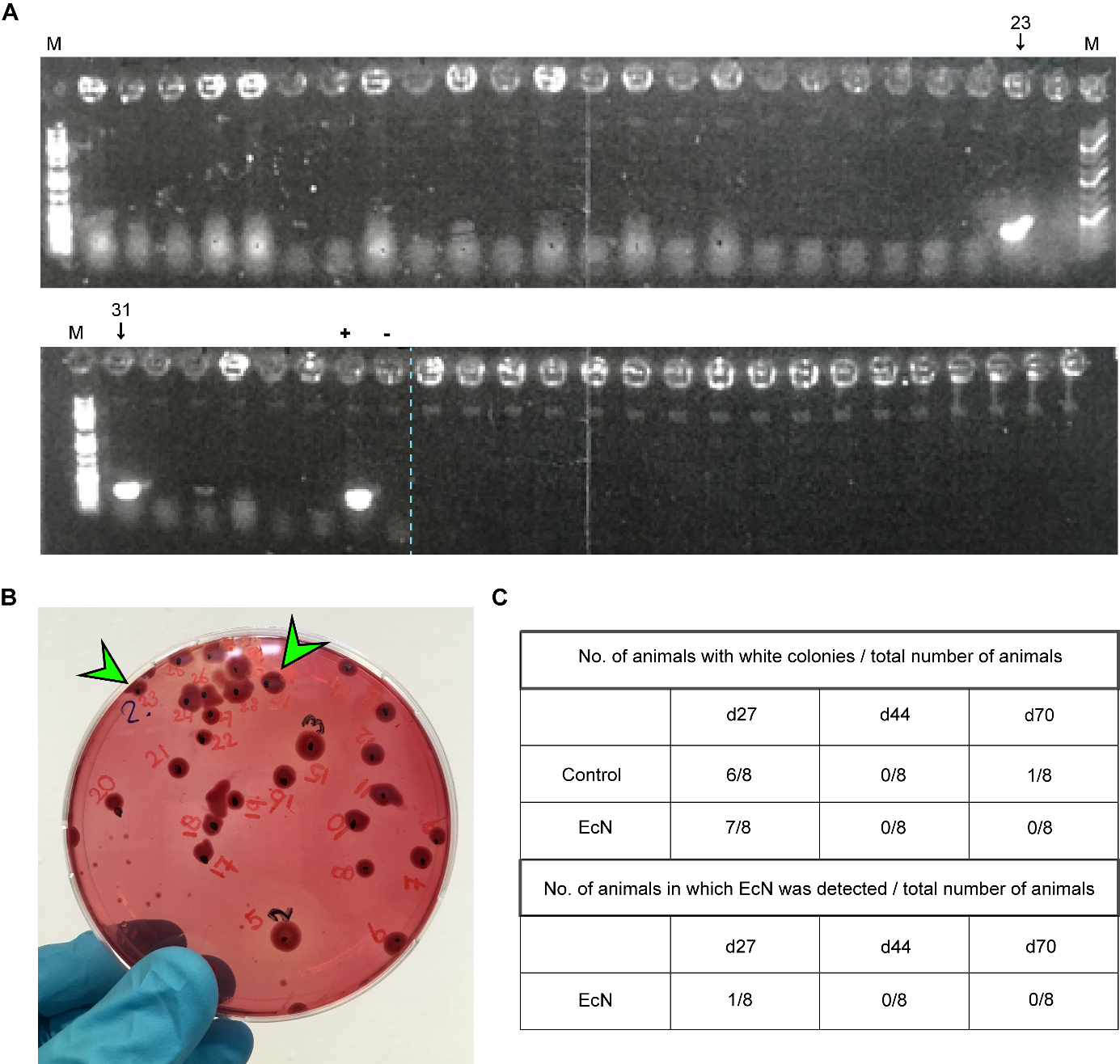


**Figure S6.** Translocation of EcN to the MLN. Detection of EcN in the MLN of EcN-treated animal using colony PCR (**A**). Arrows indicate the detection of EcN specific colonies, ‘M’; marker, + and -; positive control and negative control, respectively. Bacteria from the MLN grown on MacConkey agar (**B**). Arrows (in green) point at EcN specific colonies that were identified by colony PCR. Bacterial translocation to the MLN between the treatment groups over time (**C**). Pre-weaning (day 27) off-white opaque colonies were detected in the majority of both control and EcN-treated animals, which likely represent *Salmonella* colonies. EcN specific colonies were only detected on day 27 and in a single animal.


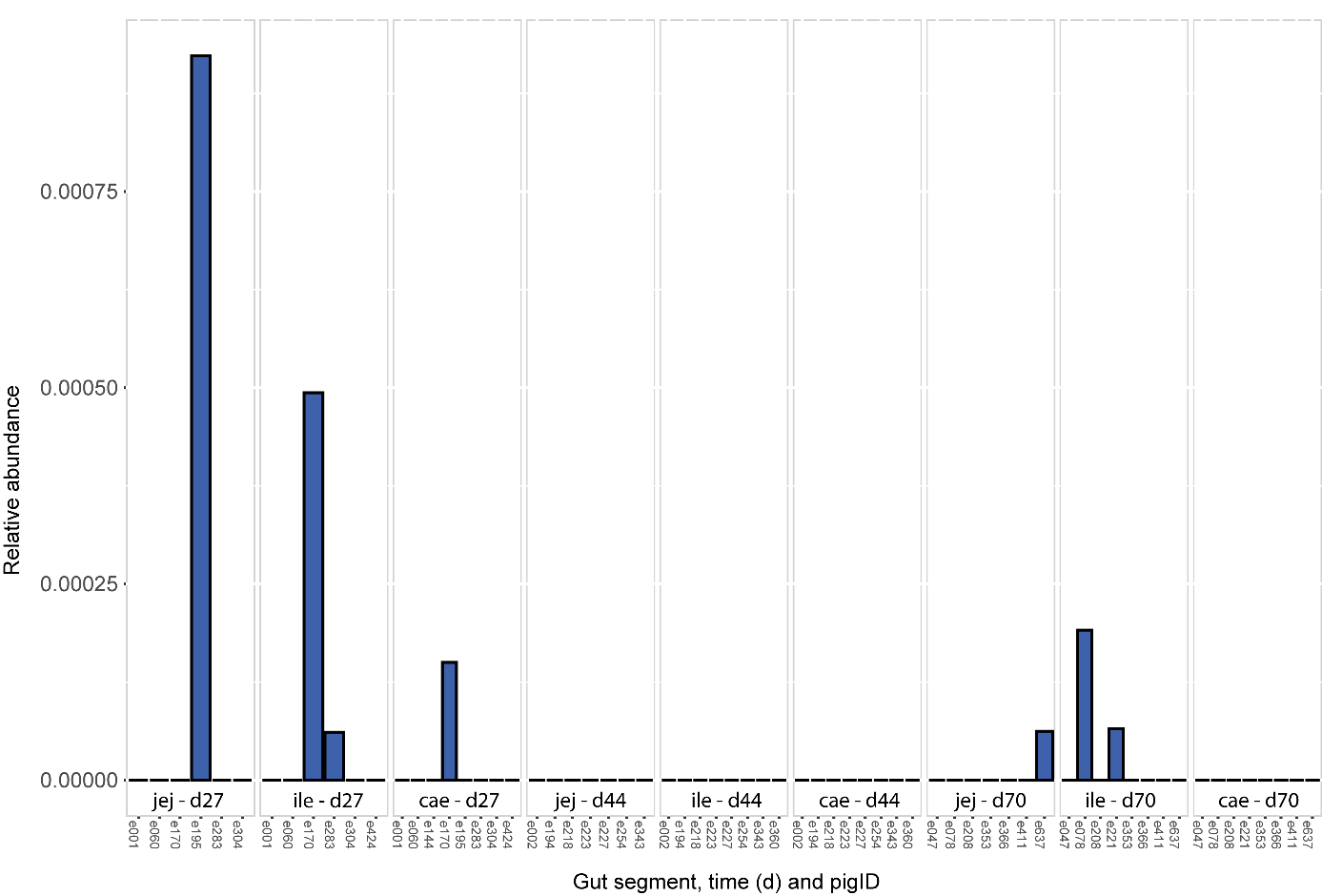


**Figure S7.** Presence of EcN in digesta of different gut segments (jejunum; jej, ileum; ile and caecum; cae) from EcN-treated pigs over time, sacrificed at the indicated days (d; day). Data is presented as the relative abundance of the EcN-specific ASV, as calculated from the NGS dataset.


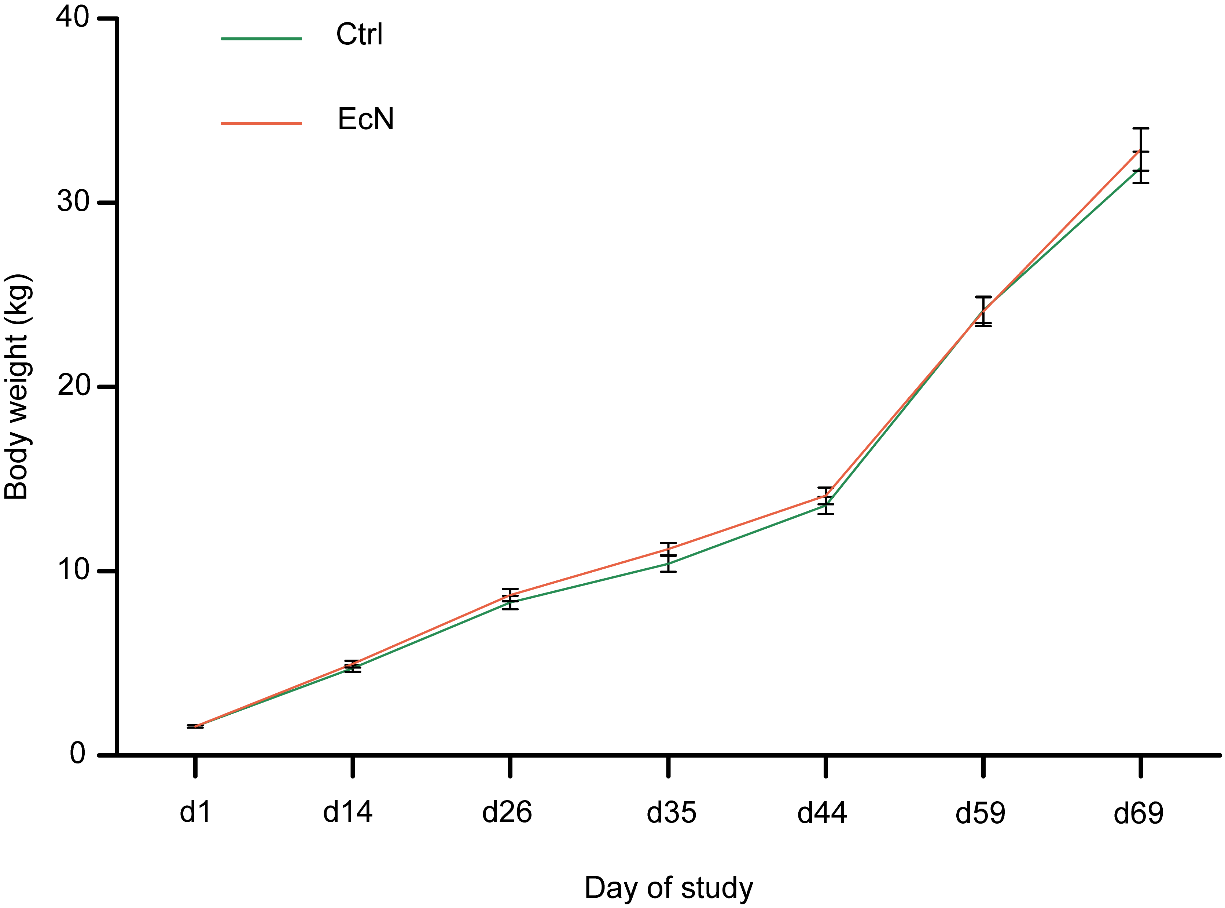


**Figure S8.** Body weight (kg) over time between the treatment groups. Animals included in this graph were followed during the entire study (n=16 per treatment group). At the start of the study, groups were made as equal as possible in terms of body weight, time of birth and sow parity. Data are shown as the means ± the standard error of the mean (SEM). No significant differences were observed between the treatment groups.


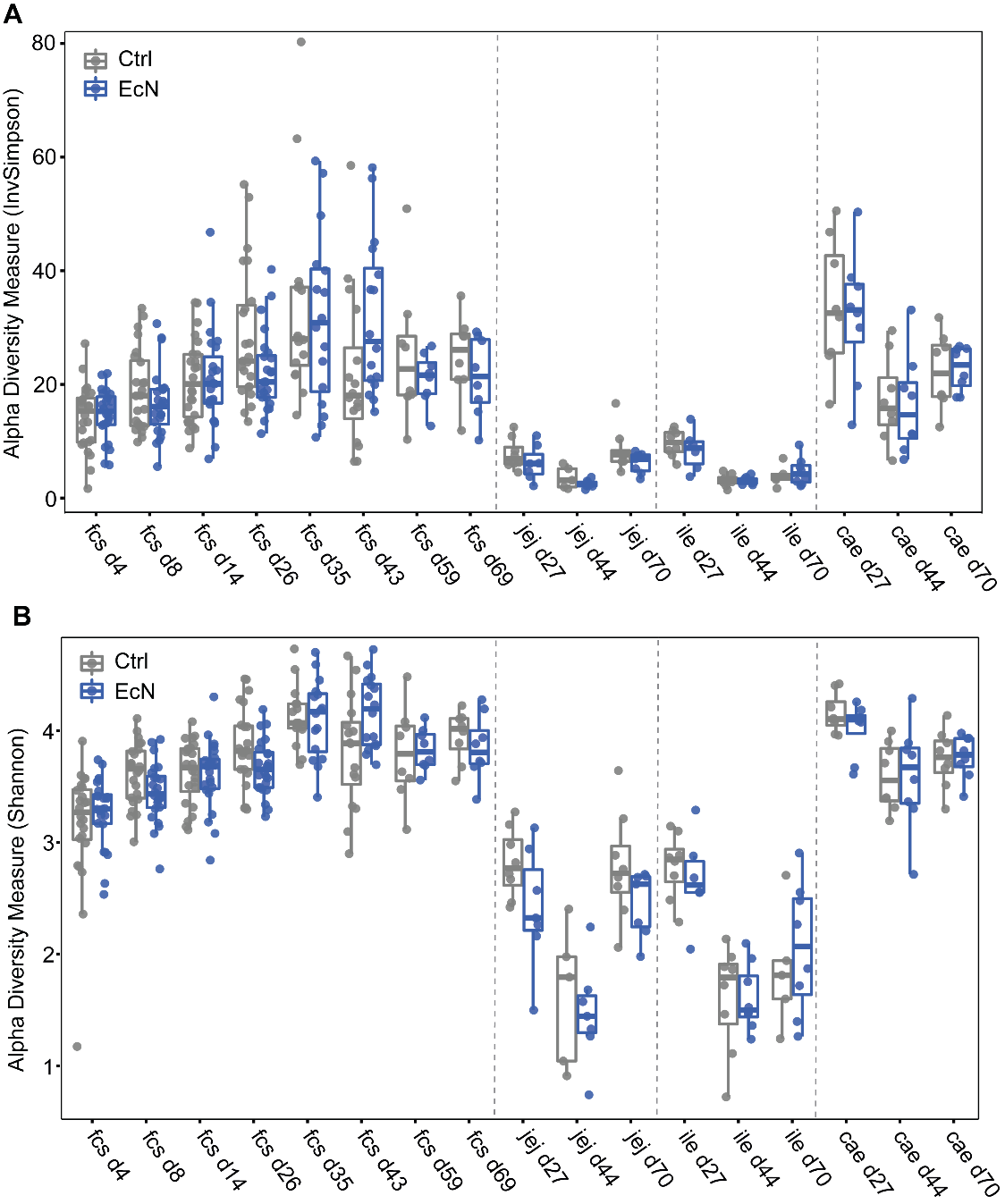


**Figure S9.** Alpha-diversity of porcine gut microbiota in faeces and digesta between treatments over time. Every point represents an individual animal with the control animals in grey and the EcN-treated animals in blue. InvSimpson (**A**) and Shannon (**B**) diversity values are presented by sampling time point (day 4 – day 70), and by faeces (fcs) or gut segment (jej; jejunum, ile; ileum, cae; caecum).


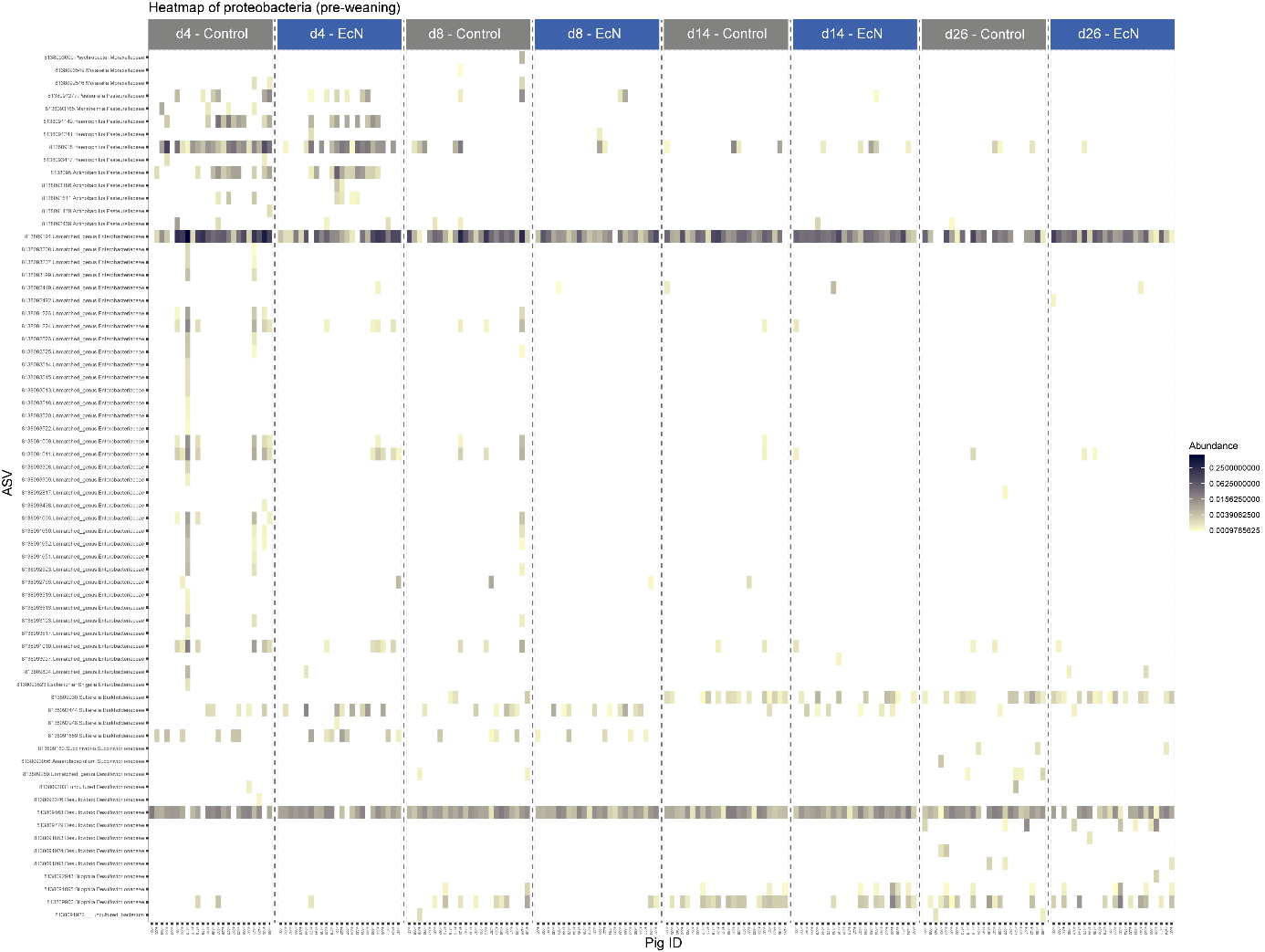


**Figure S10.** Heatmap of the relative abundance of all ASVs belonging to the phylum Proteobacteria in pre-weaning faecal samples over time. Each time point includes 24 faecal samples per treatment group (8 control pens and 8 EcN pens, 3 animals per pen). This figure shows that especially ASVs in the family of *Enterobacteriaceae* contribute to a higher number of Proteobacteria ASVs in the control group.


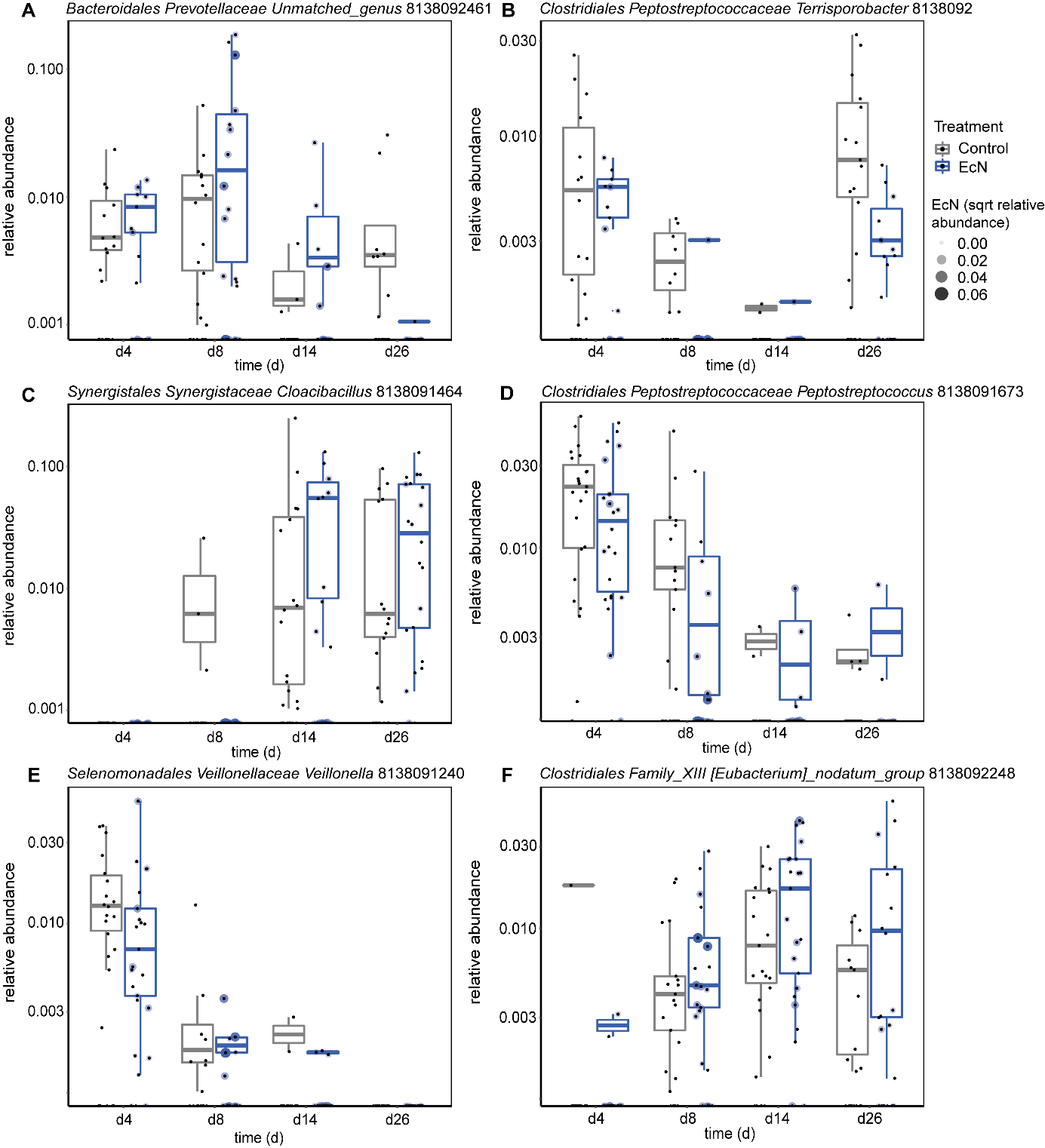


**Figure S11.** Identification of differentially abundant genera in faeces during the pre-weaning period (**A-F**). Data are presented as relative abundances (by fraction) over time. Shown genera resulted from comparing control animals (grey) to EcN-treated animals (blue) using a GAMLSS model and after ajdustment for multiple testing (p.adjust < 0.05). Every point represents an single animal, and the size of the points gives the relative abundance (sqrt) of EcN detected in this individual.


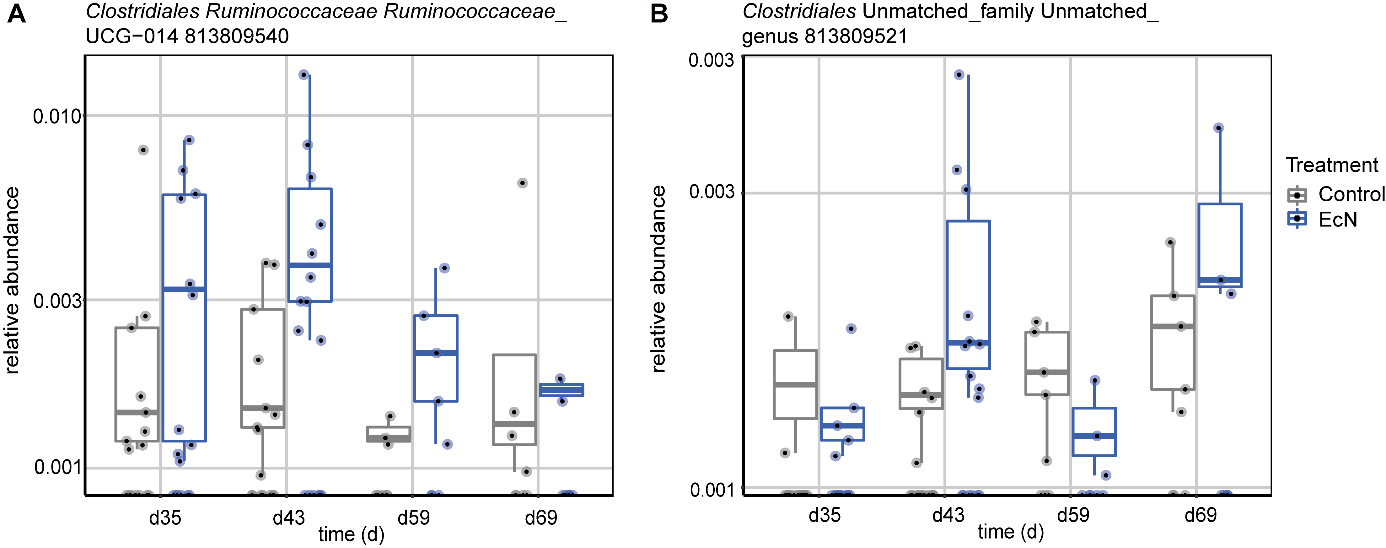


**Figure S12.** Identification of differentially abundant genera in faeces during the post-weaning period (**A, B**). Data are presented as relative abundances (by fraction) over time. Shown genera resulted from comparing control animals (grey) to EcN-treated animals (blue) using a GAMLSS model and after adjustment for multiple testing (p.adjust < 0.05). Every point represents a single animal, and the size of the points gives the relative abundance (sqrt) of EcN detected in this individual.


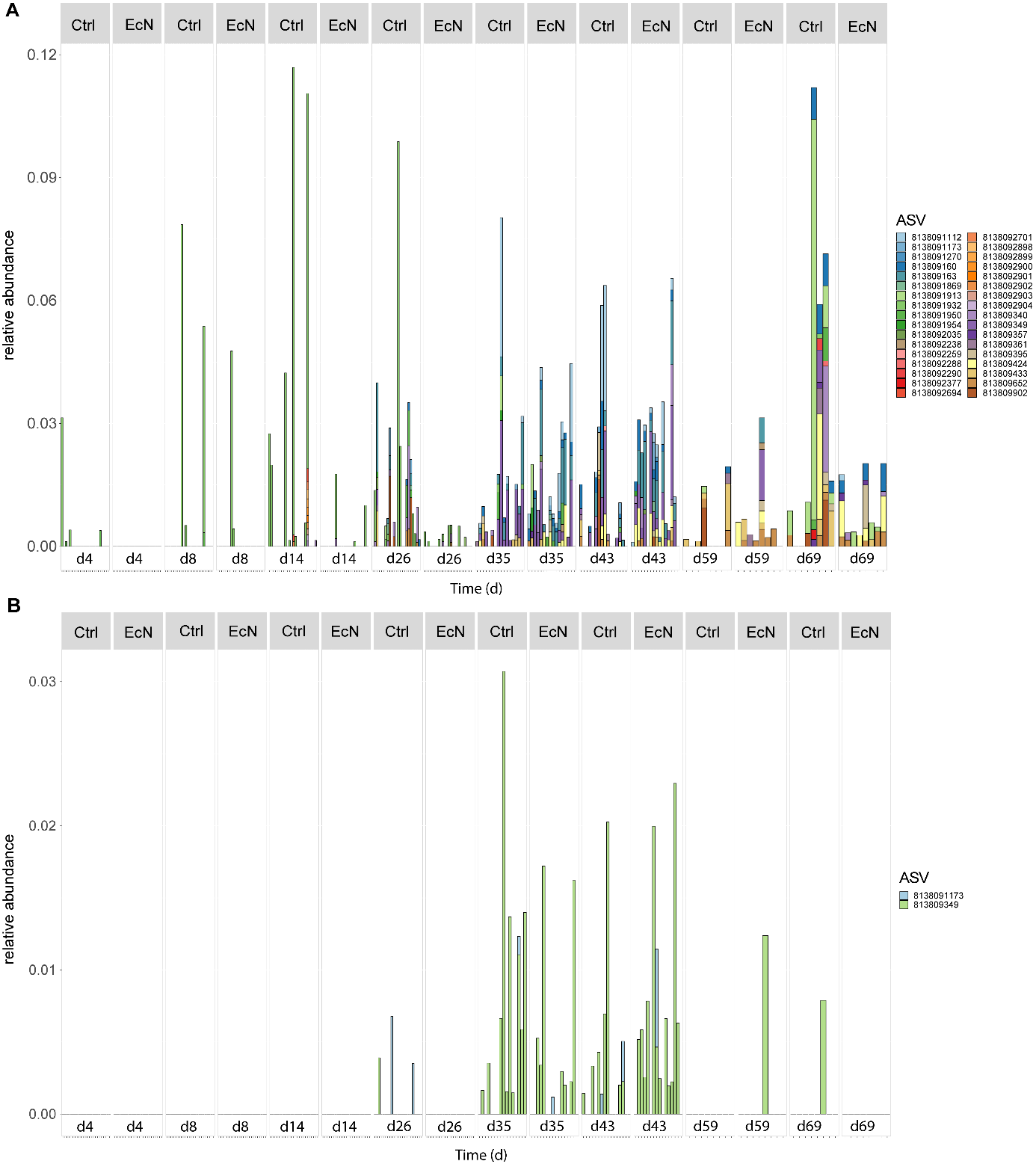


**Figure S13.** Relative abundance of all ASVs within the *Treponema_2* genus over time in faecal samples (**A**), and the relative abundance of the two ASVs that both correspond to the sequence of the earlier reported invasive *Treponema* species, namely *Candidatus Treponema suis* (**B**). This *Treponema* ASV is especially abundant in the immediate post-weaning period up to two weeks post-weaning (d35 and d43). Samples are ordered chronologically and are separated by treatment group (Ctrl versus EcN).


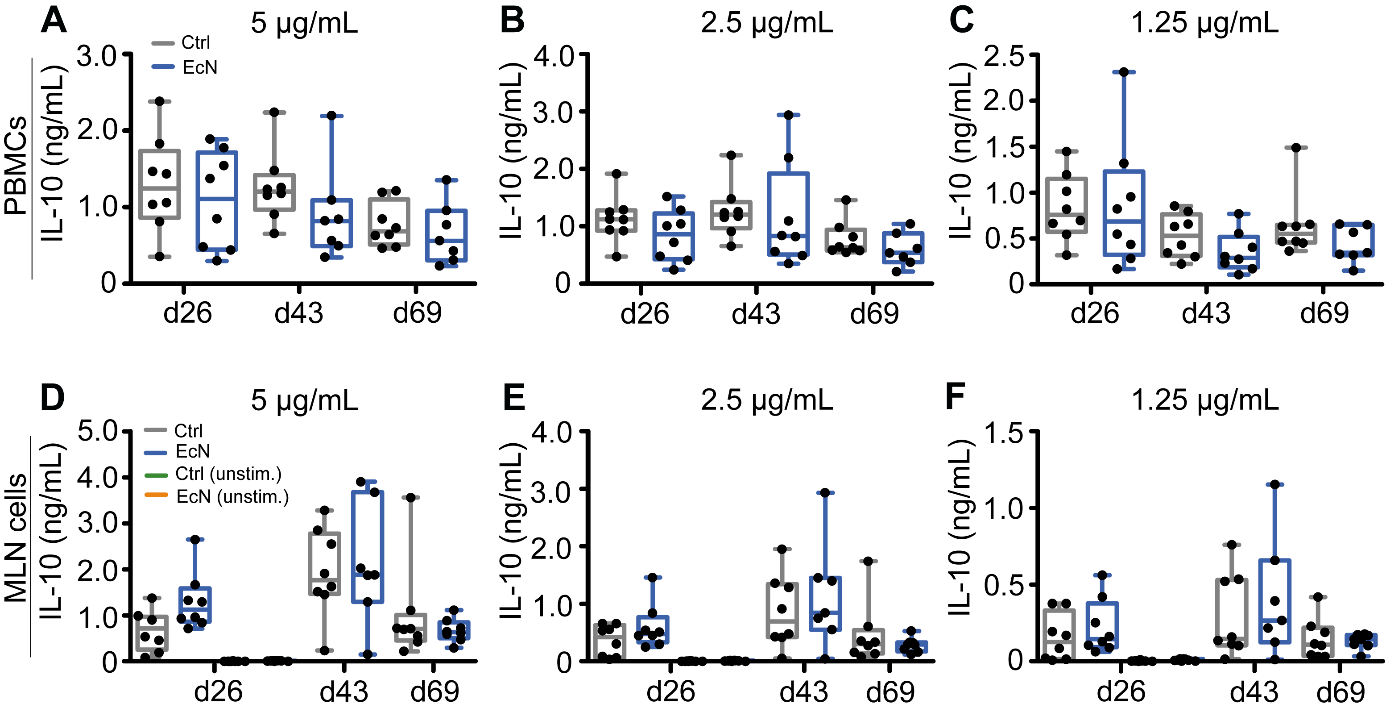


**Figure S14.** IL-10 production by *ex vivo* stimulated PBMCs and MLN cells. PBMCs (**A-C**) and MLN cells (**D-F**) were stimulated with 5 µg/mL, 2.5 µg/mL, 1.125 µg/mL of ConA or left unstimulated (cell culture medium only) for 24 h. Every point represents a single animal from a different pen (n = 7 or 8 per treatment group). No significant differences were found between the treatment groups. Normal distribution and equal variances of data were checked and log-transformed when necessary.
